# Metal‐Organic Framework‐Based Supercapacitors: A Comprehensive Review

**DOI:** 10.1002/advs.202519519

**Published:** 2026-01-27

**Authors:** Swapnajit V. Mulik, Mrunal M. Patil, Sonali P. Sadavar, Sagar D. Delekar, Rajendra K. Bordia, Dillip K. Panda

**Affiliations:** ^1^ Department of Chemistry Shivaji University Kolhapur Maharashtra 416004 India; ^2^ Department of Chemistry Dattajirao Kadam Arts Science and Commerce College, Ichalkaranji Maharashtra 416115 India; ^3^ Department of Materials Science and Engineering Clemson University Clemson SC 29634 USA

**Keywords:** metal‐organic frameworks (MOFs), nanostructures, supercapacitors (SCs)

## Abstract

Metal‐organic frameworks (MOFs) are considered to be one of the important class of materials for energy storage devices, particularly for supercapacitors (SCs), having tunable pore sizes, large surface area, and the possibility of numerous organic‐inorganic compositions. This review covers various synthesis strategies as well as fundamental properties of MOFs benefiting SCs. The MOFs and their derived materials, such as metal oxide nanostructures, binary metal oxides, porous carbon, and their functional composites, are highlighted with respect to the SC applications. Furthermore, the review emphasizes a collection of promising data to revolutionize the design of MOF‐based porous electrode materials, enhancing SCs performance. In addition, the challenges and future trends of using MOFs and MOF derivatives as electrode materials for SCs in the real world are provided with relevance and practical applications. Also, several aspects providing valuable insights for the development of the next generation of high‐efficiency SCs by shedding light on the future applications are briefly discussed.

## Graphical abstract

Overview of Metal‐Organic Frameworks (MOFs) derived electrode materials for supercapacitors. Illustration of redox reactions, conductivity enhancement, and structural stability during charge‐discharge cycling. Emphasis on synergistic effects due to surface area, porosity leading to improved electrochemical performance and energy storage efficiency.

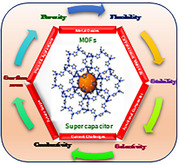

## 1 Introduction

**Figure 1:**
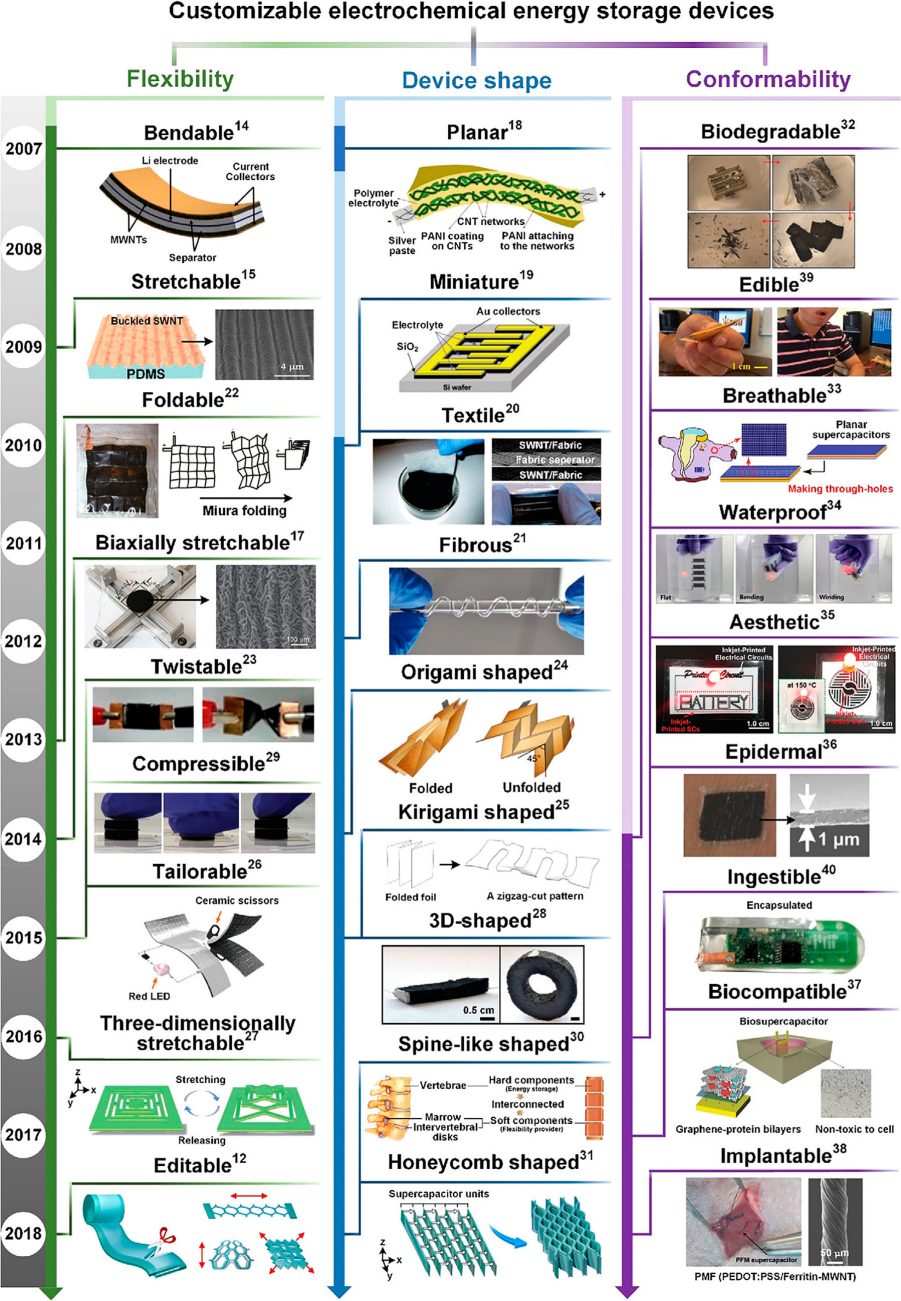
The initial decade of developing customizable electrochemical energy storage devices. Reproduced with permission.^[^ ^9^ ^]^ Copyright 2019, American Chemical Society.

The increasing population is creating strain on traditional energy resources, demanding advanced energy conversion and storage technologies.^[^ ^1^, ^2^ ^]^ It is crucial to store renewable energy adequately to meet the needs of the developing world.^[^ ^3^ ^]^ In connection with it, different energy‐storing devices have been explored, including electrolytic capacitors, electrochemical supercapacitors (SCs), fuel cells, lithium‐ion batteries, etc.^[^ ^4^, ^5^ ^]^ Particularly, SCs are under prime focus because their performance lies between batteries and conventional capacitors.^[^ ^6^ ^]^ In addition, SCs surpass the collective drawbacks of batteries and capacitors by exhibiting rapid charge–discharge capabilities (seconds), good stability, excellent rate performance, low maintenance costs, and better power density (Pd) as well as energy density (Ed).^[^ ^7^, ^8^ ^]^ But when we consider the Ed and Pd of the batteries and capacitors, respectively, SCs are still way behind. So, exploring the advanced electrode active materials for SCs is also imperative to withstand rigorous operational demands while maintaining the properties along with life span, increased mechanical flexibility, high efficiency, and reliability (**Figure** **1**).^[^ ^9^ ^]^

Conventional electric double‐layer capacitors (EDLCs) utilize carbonaceous materials, which demonstrate high Pd with long life due to the electrostatic way of energy storage mechanism.^[^ ^10^ ^]^ But such mechanisms limit EDLCs in terms of Ed.^[^ ^5^, ^11^ ^]^ However, in contrast to EDLCs, pseudocapacitors utilize redox‐active materials such as conducting polymers and transition metal oxides (TMOs), offering higher Eds.^[^ ^12^ ^]^ TMO‐based materials usually suffer from challenges such as low conductivity as well as cycle stability.^[^ ^13^ ^]^ The collective drawbacks of both EDLCs and pseudocapacitors are mitigated by hybridizing the electrostatic mechanism of EDLCs with the Faradic mechanism of the pseudocapacitors.^[^ ^14^, ^15^ ^]^ Hybrid SCs (HSCs) consisting of carbonaceous materials with redox‐active materials achieve superior energy storage performance.^[^ ^7^ ^]^

But still, these materials are obscured in performance, as most of the material acts as dead volume due to limitations in the diffusion of electrolyte ions.^[^ ^16^ ^]^ However, it is the need of the hour to have advanced porous materials with controlled pore size and volume to achieve desirable SC performance. Developing advanced functional materials with enhanced electrochemical properties, such as high stability, better conductivity, and tunable pore size and volume, is crucial for next‐generation high‐performance energy storage applications.^[^ ^17^, ^18^ ^]^

Recently, metal‐organic frameworks (MOFs) have gained immense attention due to their fascinating structures and diverse physicochemical properties.^[^ ^18^ ^]^ MOFs, a novel class of porous crystalline materials composed of metal ions or clusters and organic linkers, are highly valued for their exceptionally high surface areas, tunable pore sizes, and versatile structures.^[^ ^19^, ^20^ ^]^

The multifunctionality and versatility of MOFs drive significant interest among researchers for developing numerous MOFs for applications in different sectors.^[^ ^21^ ^]^ The vast array of metal ions and organic linkers allows for limitless combinations of MOFs, tailoring them for specific electrochemical requirements. However, their inherent poor electrical conductivity and degradation in electrolytes present challenges for direct use.^[^ ^22^ ^]^ To overcome these limitations, combining MOFs with conductive materials like carbon or conductive polymers can enhance their electrochemical performance.^[^ ^23^, ^24^ ^]^ Additionally, MOFs can serve as sacrificial templates to achieve uniformly distributed porous carbon, metal oxide/hydroxide, metal sulfide, and metal phosphide nanostructures through various chemical and thermal treatments.^[^ ^18^, ^25^ ^]^ The MOF‐derived material mitigates some of the drawbacks of the parent MOFs, which usually showcase poor electrical conductivity along with an average life span due to degradation in electrolytes.^[^ ^26^ ^]^

Recent research has focused on designing and synthesizing a variety of MOFs and exploring their potential as energy storage materials. The present review comprehensively explores recent studies of MOFs describing different synthesis strategies to achieve MOFs and their functional materials with various physicochemical properties benefiting SC applications. It emphasizes the potential of various MOFs‐derived bare as well as binary metal oxides, carbonaceous materials, and their functional composites, to enhance SC performance. It also addresses current challenges, future trends, and the practical applications of MOF‐derived materials, highlighting strategies for designing next‐generation SCs.

## 2 Unveiling the Fundamentals of Metal‐Organic Frameworks

MOFs are crystalline porous coordination polymer materials made up of secondary building units, or central metal units, connected to organic linkers via coordination covalent bonds.^[^ ^27^ ^]^ MOFs can be synthesized with desired one, two, and three dimensions by properly optimizing the synthesis conditions as well as specifically choosing organic and inorganic units having specific size, geometry, and functionality.^[^ ^28^ ^]^ This flexibility makes MOFs one of the most adaptable materials for numerous applications, such as gas sensing, biomedical applications, and in the energy sector for energy conversion and storage.^[^ ^29^, ^30^ ^]^ A uniform distribution within the framework ensures tailored properties for diverse applications.^[^ ^31^ ^]^

**Figure 2:**
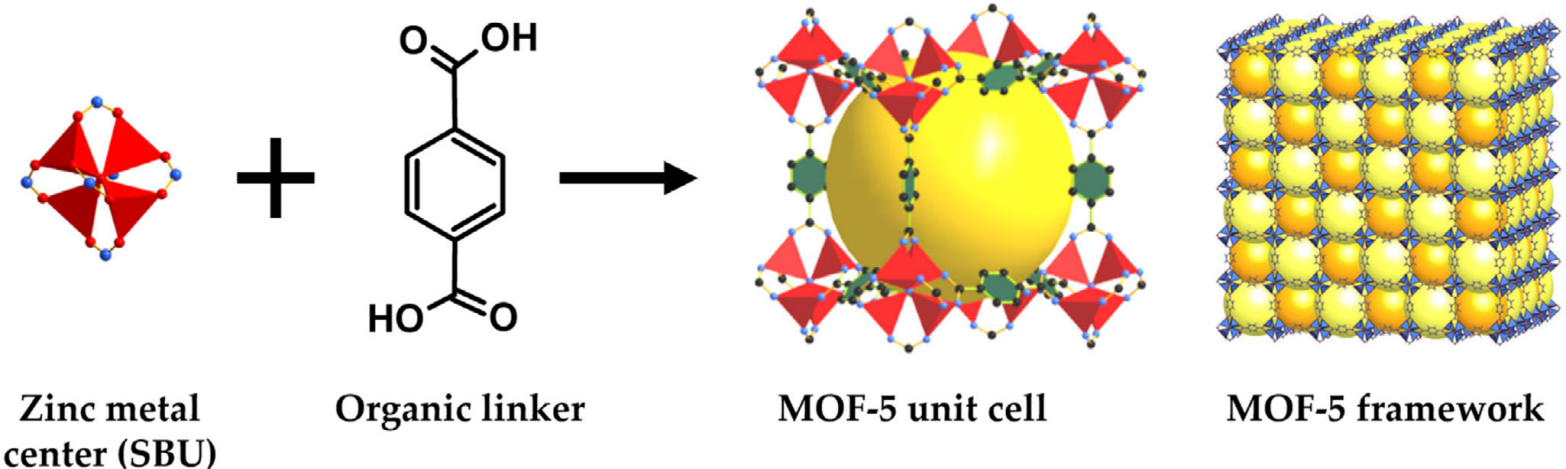
Illustration of the synthesis of MOF‐5. Reproduced with permission.^[^ ^33^ ^]^ Copyright 2016, MDPI.

Structural and chemical elements influencing electrochemical processes are evaluated by the regulation of pore size and chemical environment in isoreticular MOFs.^[^ ^32^ ^]^ Thus, such optimization has evolved the MOFs from early coordination polymers to today's well‐structured and highly functional materials. The first porous coordination polymers were synthesised by using zinc as a metal ion and BDC, i.e., 1,4‐benzenedicarboxylate (terephthalic acid) as a linker.^[^ ^33^ ^]^ This synthesized porous coordination polymer featured a 3D cubic structure, forming a robust framework (**Figure** **2**).^[^ ^33^ ^]^ The scaffolding‐like structure of MOF‐5 and its derivatives resulted in exceptionally high surface areas, ranging from 2500 to 3000 m^2 ^g^−1^ with high thermal stability ranging between 300 and 400 °C.^[^ ^34^ ^]^ These physicochemical properties of MOF‐5 laid a foundation stone for designing and tuning the chemical properties, pore sizes, and particle dimensions of coordination polymers by changing the centre metal ion and linker. Three years after the discovery of MOF‐5, Yaghi and his team synthesized a set of MOFs based on MOF‐5 geometry by varying the functional groups (halogens, alkyl, hydroxyl, and amino) on the ligand.^[^ ^35^ ^]^ These Isoreticular MOFs (IRMOFs) demonstrated high surface areas, tuneable pore sizes, and diverse functionalities, opening new avenues for synthesizing various coordination polymers.^[^ ^36^ ^]^ Yaghi's landmark studies established MOFs, serving as an exceptional material for making highly porous and conductive derivatives with longevity in industrial applications. For this milestone, Yaghi, along with S. Kitagawa and R. Robson, received the 2025 Nobel Prize in Chemistry, demonstrating fundamental scientific vision transformed into real‐world technologies.^[^ ^37^ ^]^

**Figure 3:**
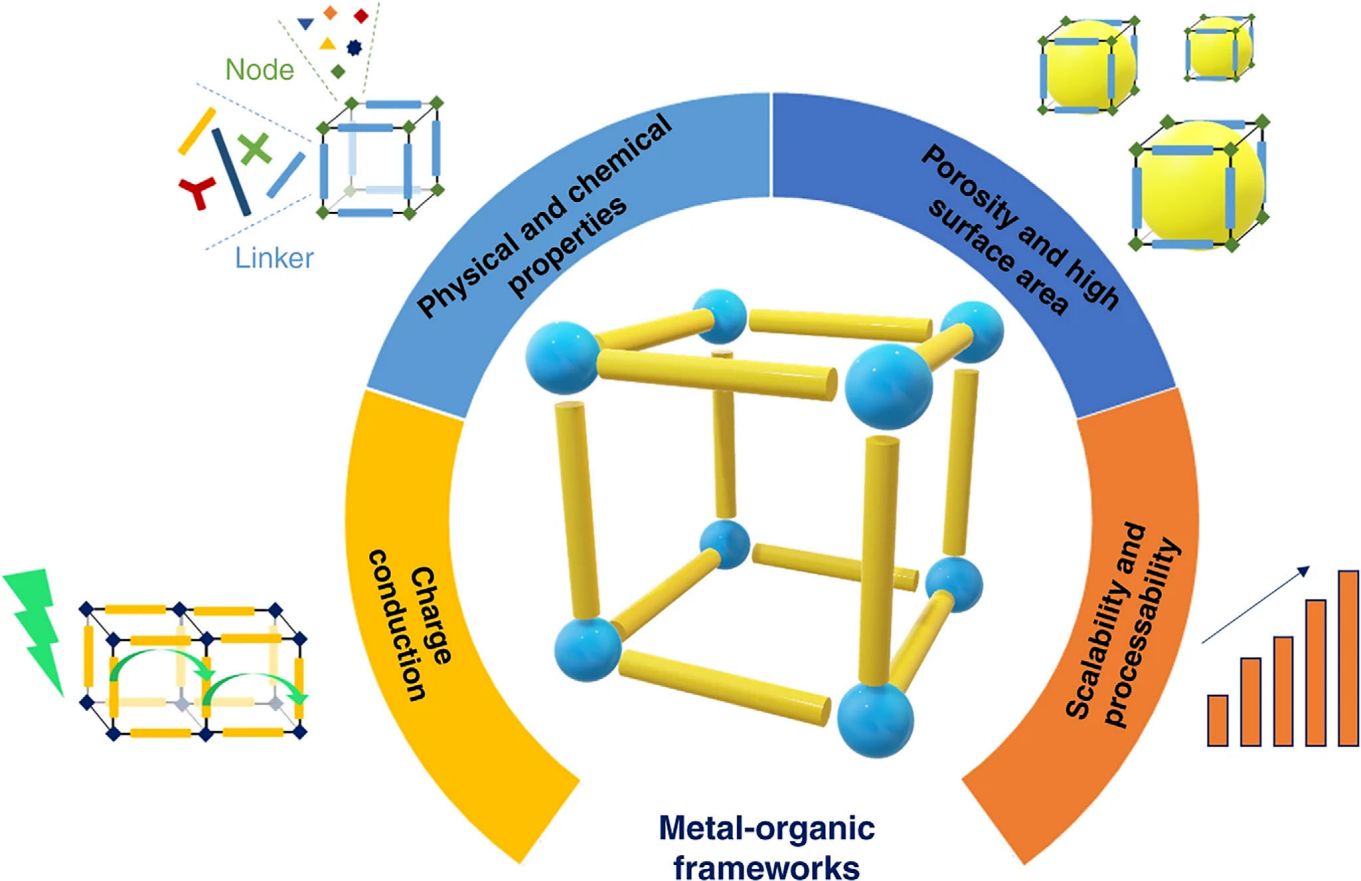
Tunable MOF attributes for electrochemical applications. Reproduced with permission.^[^ ^38^ ^]^ Copyright 2019, Nature.

Hierarchically porous MOFs, characterized by various pore sizes that may be microporous/mesoporous/macroporous, create strategic channels and spaces within pores that facilitate diffusion.^[^ ^38^, ^39^, ^40^ ^]^ In a recent study by Salunkhe et al. developed 3D porous carbon was developed from polystyrene spheres coated with ZIF‐8 MOF.^[^ ^41^ ^]^ The carbonized nanocomposite (NCs) showcases a hierarchical porous structure with a large surface area of 1067 m^2^ g^−1^ and a significant pore volume (1.12 cm^3^ g^−1^), providing rapid ion diffusion and electrolyte penetration. This capability of MOFs to be engineered with specific surface areas makes them highly significant. Thus, MOFs can be processed and functionalized to impart new physical and chemical properties (**Figure** **3**).^[^ ^38^ ^]^ MOFs with better porosity led to better exploration of redox‐active sites, mitigating the use of traditional inorganic solids as a promising material for electrochemical applications. **Table** **1** highlights various MOF‐based materials with their pore volume and surface area details. Early developed MOFs like Zn_4_O(BDC)_3_ and Cr_3_(O, OH) (H_2_O)_2_O (BDC)_3_·nH_2_O possess surface areas of 3800 and 4100 m^2^ g^−1^, respectively, opening avenues for the development of highly porous materials.^[^ ^42^, ^43^ ^]^ The advancements in the design of linkers, specifically the use of aromatic ligands like Tetrakis (4‐carboxyphenyl) porphyrin (TCPP), 1,3,6,8‐Tetrakis (p‐benzoate) pyrene (TBAPy), enable the achievement of ultrahigh surface areas within NU‐1501 (7310 m^2^ g^−1^) and DUT‐60 (7840 m^2^ g^−1^).^[^ ^44^, ^45^, ^46^ ^]^ These massive improvements in both surface area and pore volume signify the evolution of MOFs chemistry for diverse applications.

**Table 1:** Representative MOFs with porosity parameters.

Name of MOFs	Linkers	Composition	BET surface area [m^2^ g^−1^]	Pore volume [cm^3^ g^−1^]	Year reported	Refs.
MOF‐5	Benzene‐1,4‐dicarboxylate (BDC)	Zn_4_O(BDC)_3_	3800	1.55	1999	[42]
MIL‐101	Benzene‐1,4‐dicarboxylate (BDC)	Cr_3_(O, OH) (H_2_O)_2_O (BDC)_3_·nH_2_O	4100	2.0	2005	[43]
MOF‐205 (DUT‐6)	Tetrakis (4‐carboxyphenyl) porphyrin (TCPP)	Zn_4_O(TCPP)	5900	2.42	2009	[69]
MOF‐200	4, 4′, 4′“, 4′”'‐Benzene‐1,2,4,5‐tetrayltetrabenzoate (BTB)	Zn_4_O(BTB)_3_	4530	3.59	2010	[70]
NU‐100	1,3,6,8‐Tetrakis(p‐benzoate)pyrene (TBAPy)	Cu_4_(µ_4_‐O) (H_2_O)_4_ (TBAPy)	6143	2.82	2010	[71]
DUT‐32	4,4′‐biphenylene dicarboxylic acid (bpdc)^2−^ 4,4′,4′′‐[benzene‐1,3,5‐triyltris(carbonyl imino)]tris‐benzoate) (btctb)^3−^	Zn_4_O(bpdc) (btctb)_4/3_	6411	3.16	2014	[72]
NU‐1103	Tetrakis(4 carboxyphenyl)porphyrin (TCPP)	Zr_6_O_4_(OH)_4_ (TCPP)_3_	6550	2.91	2015	[44]
DUT‐60	1,3,6,8‐Tetrakis(p benzoate)pyrene (TBAPy)	Zn_4_O(µ_4_‐O) (TBAPy)_2_	7840	5.02	2018	[45]
NU‐1501	Triptycene, µ_3_‐oxo‐centred trinuclear clusters	Al_3_(µ_3_‐O) (H2O)_2_(OH) (PET)	7310	2.91	2020	[46]

The unique charge conduction properties are rooted in their distinct structural and chemical compositions.^[^ ^47^ ^]^ MOFs can conduct charge through several mechanisms. First, their electronic structure allows for the delocalization of electrons through metal‐to‐ligand charge transfer and vice versa.^[^ ^48^ ^]^ Some MOFs exhibit redox‐active metal centres or organic ligands capable of reversible redox reactions, often known as redox‐active MOFs.^[^ ^49^ ^]^ Such redox‐active MOFs further improve the efficiency of electrochemical charge storage devices.^[^ ^49^, ^50^ ^]^ Additionally, the presence of guest molecules such as tetrathiafulvalene (TTF), ferrocene, etc., within MOFs can influence their charge conduction properties by altering the electronic structure of the framework or redox characteristics.^[^ ^51^, ^52^ ^]^ Li et al. encapsulated benzo‐12‐crown‐4‐ether (BCE) into an anionic ZIF‐7. A bottle‐around‐ship strategy was utilised to synthesize BCE@ZIF‐7, in which BCE molecules were incorporated within the micropores.^[^ ^53^ ^]^ Further linking this core–shell material with metal cations such as Li^+^, Mg^2+,^ and Al^3+^ resulted in the M@BCE@ZIF‐7 composite. This hybrid material showed tunable surface charge properties, ion selectivity, and induced mixed conductivity, involving pathways for ionic or protonic charge transport.^[^ ^47^, ^54^ ^]^

Various researchers have incorporated metal nanoparticles (NPs) as guest species to enhance the electrical conductivity of different MOFs.^[^ ^55^, ^56^ ^]^ Han et al. demonstrated the synthesis of the semiconducting MOFs by encapsulating silver nanoparticles (Ag NPs) within the framework.^[^ ^57^ ^]^ The cyclodextrin‐based Rb‐CD‐MOF (64) crystals adsorb silver ions when immersed in AgNO_3_ solutions. Where Ag NPs are formed by reducing AgNO_3_ suspension with hydroxide (‐OH) groups of the cyclodextrin. The electrical conductivity of MOF was observed to be raised from 6.8 × 10^−10^ S cm^−1^ to 3.1 × 10^−9^ S cm^−1^ by embedding the Ag NPs.^[^ ^57^ ^]^ The defects or disorders within MOFs structures play a crucial role in their charge conduction behaviour, offering opportunities for enhanced conductivity by creating channels for facilitating ion migration as well as offering better adsorption capacity.^[^ ^58^, ^59^ ^]^ These versatile properties position MOFs as promising materials for SC applications, where controlled charge transport is crucial for capacitive performance and functionality.^[^ ^60^ ^]^

**Figure 4:**
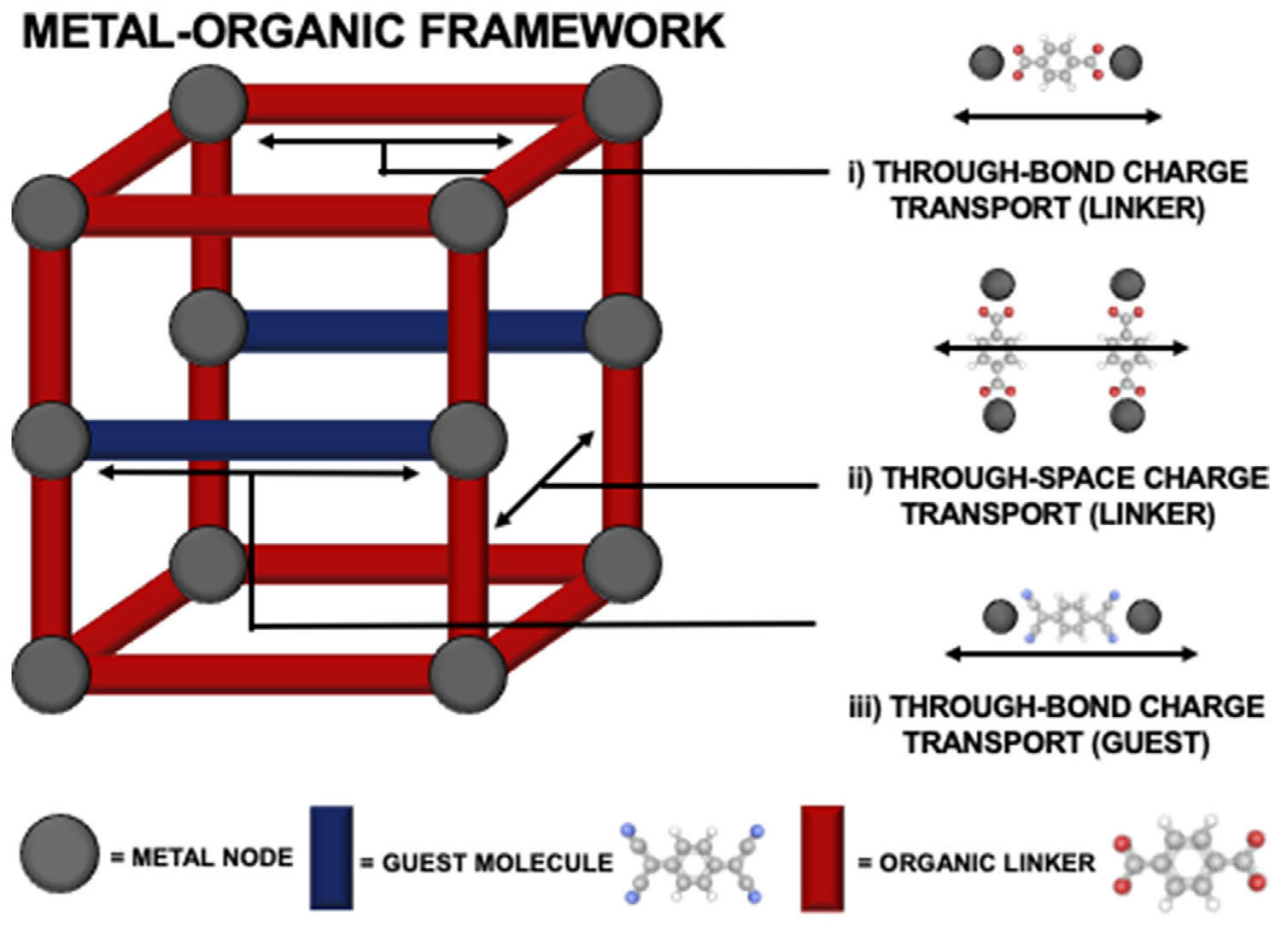
Hypothetical representation of charge transport modes in MOFs: a) Through‐bond charge transport via the organic linker, b) Through‐Space transport via the organic linker, c) Through‐bond charge transport via an organic guest molecule. Reproduced with permission.^[^ ^61^ ^]^ Copyright 2020, APL Publishing.

To achieve high conductivity in MOFs, two primary approaches are utilized, that is, through‐bond and through‐space conduction (**Figure** **4**).^[^ ^61^ ^]^ Through‐bond conduction involves the direct transfer of electrons through the chemical bonds of MOFs structure.^[^ ^62^ ^]^ This method leverages the intrinsic conductivity of the framework, where conjugated organic linkers and strong metal‐linker interactions create continuous pathways for electron delocalization.^[^ ^48^ ^]^ Additionally, redox‐active sites, due to the metal centers and the linkers, are beneficial for electron hopping.^[^ ^63^ ^]^

Hybrid materials are achieved by incorporating conductive materials like graphene, biomass‐derived nanoporous carbon, or conductive polymers with MOFs, which boost the conduction by forming composite structures.^[^ ^64^, ^65^ ^]^ In contrast, charge transfer through‐space conduction relies on non‐bonded distances within the MOFs.^[^ ^66^ ^]^ This mechanism is enhanced by incorporating conductive guest molecules, such as iodine or tetracyanoquinodimethane (TCNQ), within the MOF pores, creating charge transfer complexes that facilitate electron movement.^[^ ^67^ ^,^ ^68^ ^]^ Talin et al. reported that introducing TCNQ in the copper‐based MOF, HKUST‐1, significantly increased its conductivity from 10^−6^ to 7 S m^−1^.^[^ ^68^ ^]^ The incorporation of TCNQ molecules bridged the Cu (II) centers, making strong electronic coupling, enabling the formation of conductive pathways in the framework.

The design of the MOFs, particularly the proximity of conductive sites and the flexibility of the framework, plays a vital role in promoting charge transport through‐space conduction.^[^ ^73^ ^]^ The optimization of the size and connectivity of pores to match the mobile ions can enhance ionic conductivity, making charge transport via through‐space mechanisms. Such mechanisms are particularly effective for applications requiring high ionic mobility.^[^ ^48^ ^]^ Both approaches, through‐bond and through‐space, offer distinct pathways to tailor MOFs conductivity in various electrochemical SC devices. The electrochemical SC behavior of MOF‐based materials is significantly influenced by the composition of the electrolyte.^[^ ^74^ ^]^ Research work of Furukawa et al. illustrates the redox potential of the ferrocene/ferrocenium couple embedded inside the {[Zn(Fcdc)(bpy)](DMF)_0.5_(MeOH)_0.5_}n (where, Fcdc = 1,1′‐ferrocene dicarboxylate) shifted to a negative value when (n‐Bu_4_N)BF_4_ was substituted with (n‐Bu_4_N)NO_3_.^[^ ^75^ ^]^ Specifically, the potential was altered to 0.78 V from 0.95 V against Ag/AgCl, driven by the strong pairing of ions between NO^3−^ and ferrocenium. This pairing lowers the oxidation potential of ferrocene. Compatibility between the electrolyte and MOFs is crucial to ensure efficient ion diffusion and stability.^[^ ^76^ ^]^

The selection of a suitable electrolyte involves considering factors such as chemical stability to avoid side reactions with the MOFs. Another aspect involves charge balancing during electrochemical processes.^[^ ^74^ ^]^ The potential window of the electrolyte must align with the operational voltages of the MOFs‐based SC device to prevent the electrolyte from decomposition as well as to maintain the overall integrity of the system.^[^ ^77^ ^]^ By optimizing these interactions, one can enhance the functionality and durability of MOF‐based electrochemical devices. MOFs can go through “post‐synthetic modification” to improve their physicochemical properties by altering, swapping, or fully removing linker or node components in the framework.^[^ ^78^ ^]^ Furthermore, the MOFs surface chemistry can be altered by adjusting the crystallographic phases as well as the size.^[^ ^79^ ^]^ This versatility enables tuning of properties by having control over host‐guest chemistry.^[^ ^80^ ^]^ By using these modifications, one can optimize MOFs for enhanced performance in various electrochemical device applications.

## 3 Synthesis Techniques, Conditions, and Morphology for MOFs and Their Derivatives

### 3.1 Synthesis Techniques for MOFs and Their Derivatives

**Figure 5:**
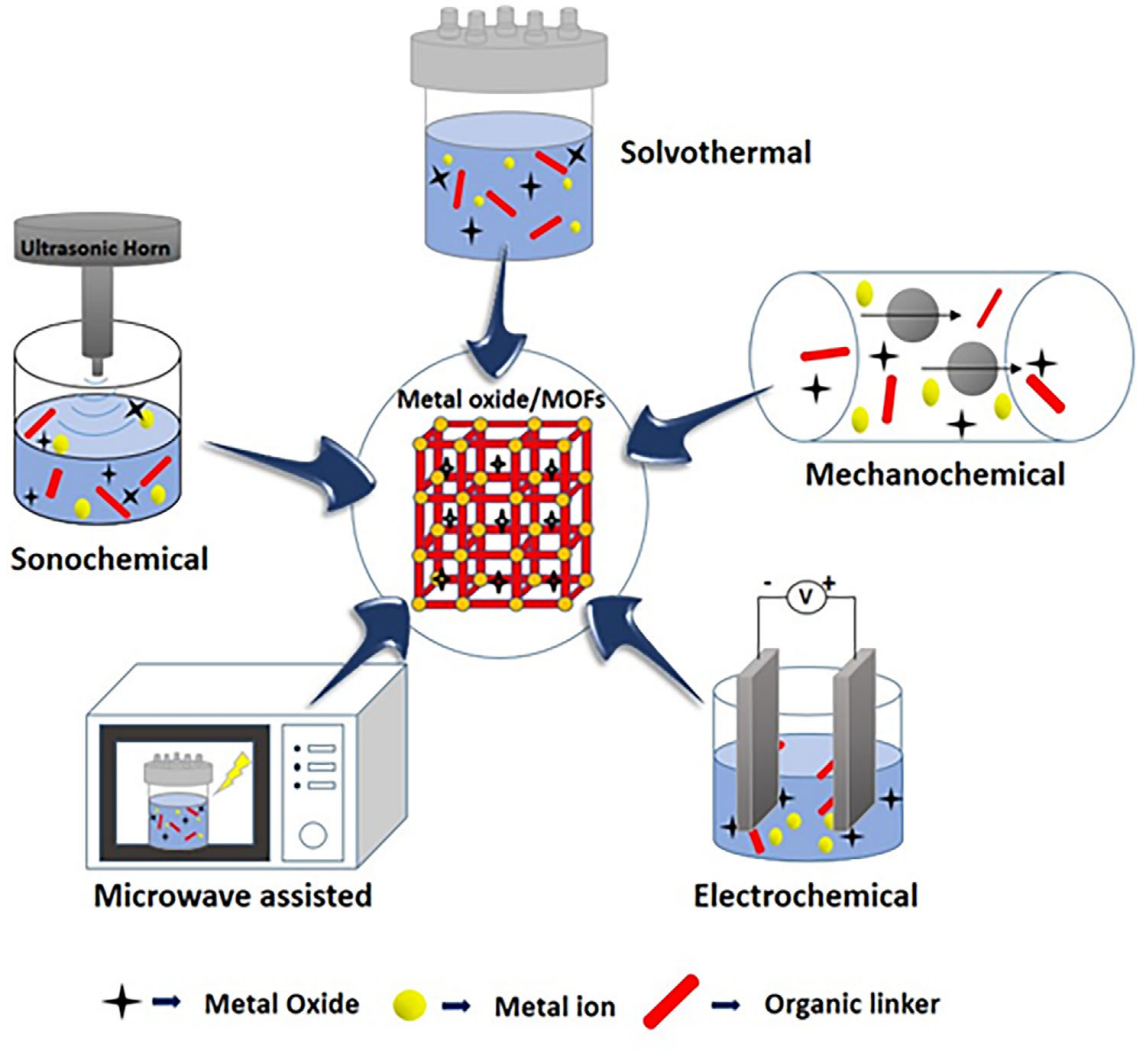
Illustration of various synthesis strategies for MOFs. Reproduced with permission.^[^ ^2^ ^]^ Copyright 2022, Elsevier.

Depending on the targeted physicochemical properties, MOFs can be synthesized using various methods.^[^ ^81^ ^]^ These methods include ultrasound‐assisted/sonochemical‐assisted, microwave‐assisted, mechanochemical, electrochemical, and hydrothermal, as well as solvothermal methods (**Figure** **5**).^[^ ^2^ ^]^ The hydrothermal or solvothermal method entails dissolving metals and organic ligands in a solvent and heating the mixture in an autoclave at 80⁰C to 260⁰C for 24 to 48 h.^[^ ^82^ ^]^ These methods have good control over the size and shape of the resulting MOFs.^[^ ^83^ ^]^

Utilizing microwave‐assisted solvothermal synthesis reduces reaction times and improves the uniformity of MOF crystals.^[^ ^84^ ^]^ Yang et al. applied the solvothermal method to develop S‐doped Ni‐MOF nanosheets that grew *in‐situ* onto a nickel foam substrate.^[^ ^85^ ^]^ The electrode fabricated using S‐doped Ni‐MOF nanosheets exhibited a specific capacitance (Cs) of 1952 mF cm^−2^ at a current density (Cd) of 1.0 mA cm^−2^. These results are attributed to the presence of abundant active sites, reduced transport barrier, and stability of the material. Further, electrochemical synthesis of porous MOFs involves anodic dissolution, where metal ions from electrodes dissolve into an electrolyte with organic ligands, initiating deposition of MOFs on the electrode surface.^[^ ^86^ ^]^ This technique offers several advantages over traditional methods, requiring milder reaction conditions, quicker synthesis, and enhanced control over MOFs properties.^[^ ^87^ ^]^ By applying voltage to an electrochemical cell, metal‐ligand complexes are formed and self‐assembled into the porous structures at the electrodes. Critical parameters like voltage, electrolyte composition, current, and electrode material etc., significantly influence the synthesis process.^[^ ^88^ ^]^ Wechsler et al. used electrophoretic deposition (EPD) to synthesize nickel hexaaminobenzene [Ni_3_(HAB)_2_ pristine MOF] as electrode material for SCs, exhibiting superior electrochemical performance, showcasing Cs of 13.64 mF cm^−2^ with retention of about 81.2% after 50000 cycles, supporting the aforementioned facts.^[^ ^89^ ^]^

Synthesis of MOFs using the mechanochemical method harnesses mechanical force to initiate chemical reactions, often by grinding solid reactants.^[^ ^90^ ^]^ This process typically employs ball‐milling and manual grinding, substantially reducing or eliminating the need for solvents, rendering it more eco‐friendly than traditional methods.^[^ ^91^ ^]^ MOF crystals are formed when the mechanical energy from grinding generates linkages between metal ions and organic ligands.^[^ ^92^ ^]^ The reaction conditions, such as grinding time and speed, can be finely tuned to optimize product yield and quality.^[^ ^93^ ^]^ This approach offers numerous advantages, including distinctly reduced reaction times, lower energy consumption, and scalability for large‐scale production. Unlike solvothermal methods, which necessitate high temperatures and pressures, mechanochemical synthesis often operates at ambient temperature and pressure, making it a more sustainable and efficient method for producing MOFs.^[^ ^94^, ^95^ ^]^ Hameed et al. utilized a mechanochemical annealing strategy to enhance the efficiency of MOFs in SCs using three different forms of metal oxides (NiCo_2_O_4_) derived from bimetallic MOFs by integrating Ni and Co metal ions by varying linkers, namely 1,3,5‐benzene tricarboxylic acid (MO‐1), 2,6‐naphthalene dicarboxylic acid (MO‐2), and 1,2,4,5‐benzene tetracarboxylic acid (MO‐3).^[^ ^96^ ^]^ The bimetallic HSCs, incorporating these metal oxides, showed pseudocapacitive behavior, reaching up to 1.5 V with a Cs of 65.6 F g^−1^, Ed of 73.83 Wh kg^−1^, and Pd of 1181.2 W kg^−1^.

The ultrasonication or sonochemical method uses ultrasonic waves to enhance chemical reactions for synthesizing MOFs.^[^ ^97^ ^]^ This process involves mixing metal salts and organic linkers and then exposing the mixture to ultrasonic waves, during which cavitation bubbles are created.^[^ ^98^ ^]^ These bubbles collapse, generating high temperatures and pressures that enhance reaction rates and bond formation. This method accelerates the synthesis of MOFs, reducing required temperatures, improving yields, and allowing better control over particle size.^[^ ^99^ ^]^ The ultrasonication method is environmentally friendly, often reducing the need for toxic solvents.^[^ ^100^ ^]^ Xiao et al. utilized the ultrasonic strategy to synthesise Ni‐MOF as the electrode material for SCs.^[^ ^101^ ^]^ This method resulted in the synthesis of Ni‐MOF with a hierarchical microblock structure in just 2 h. The resultant Ni‐MOF showcased the outstanding electrochemical performance with a Cs of 631.0 C g^−1^ at a Cd of 1.0 A g^−1^. Moreover, excellent cycling stability was observed with retention of 85.05% of initial capacitance after almost 3000 cycles. Furthermore, MOFs can also be synthesised using the microwave method. The microwave approach utilises microwave radiation to rapidly heat a mixture of metal salts and organic linkers at a desired temperature.^[^ ^102^, ^103^ ^]^ The localized and uniform heating generated by microwave irradiation accelerates the reaction kinetics, leading to a faster synthesis rate compared to conventional methods.^[^ ^104^ ^]^ Microwave heating speeds up nucleation growth and reaction kinetics during the synthesis of MOFs, and significantly reduces the reaction time.^[^ ^105^ ^]^ This method enhances phase selectivity and provides regulations on crystal size, which leads to the formation of homogeneous MOFs.^[^ ^106^ ^]^ The microwave method is scalable and can be used to produce MOFs with tunable properties by just adjusting the microwave parameters.

### 3.2 Synthesis Conditions and Morphology Optimizations

**Figure 6:**
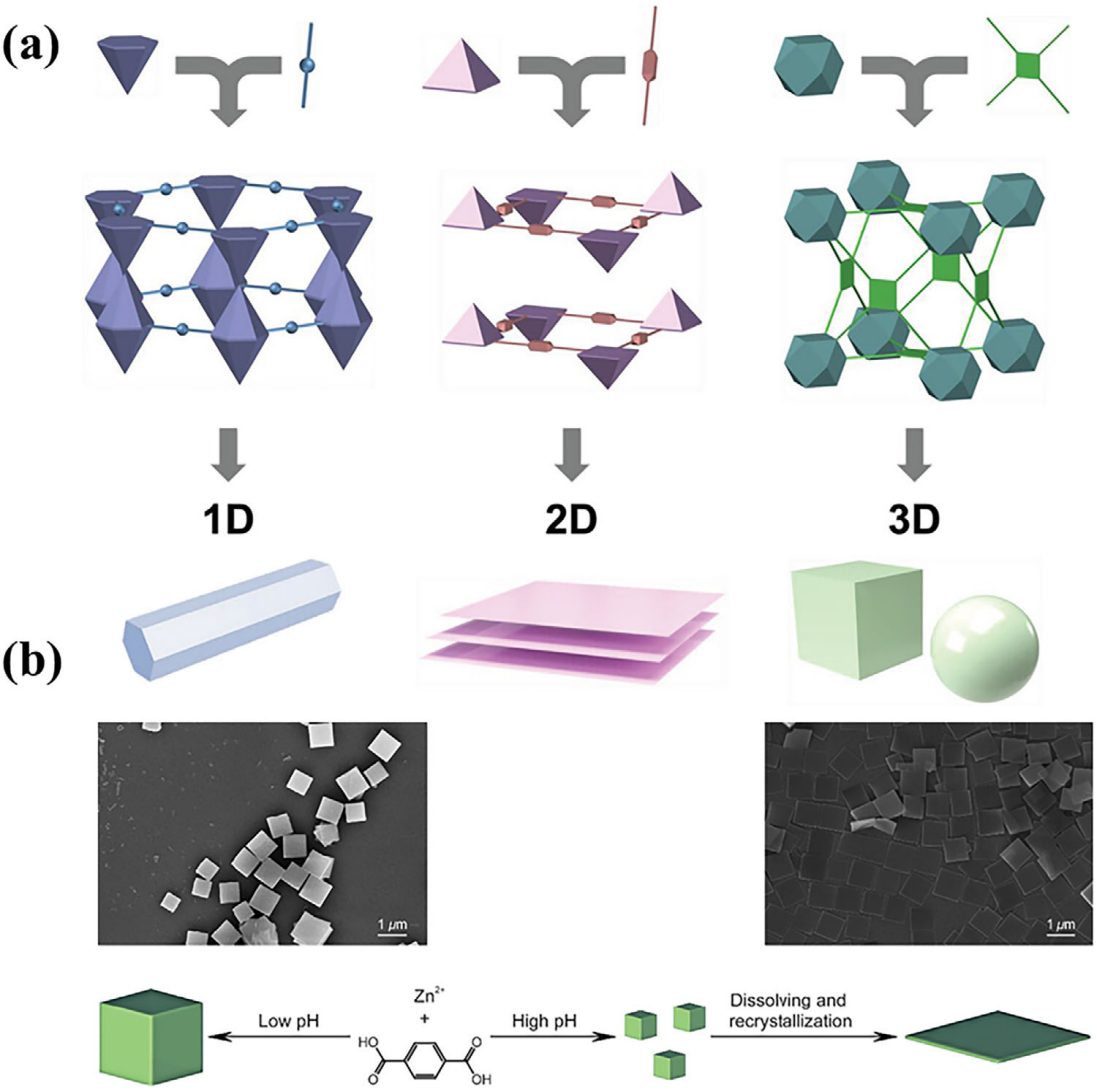
a) Demonstration of how topological driving forces influence morphological results. Reproduced with permission.^[^ ^107^ ^]^ Copyright 2023, Wiley. b) Diagrammatic representation of the evolution of MOF‐5 cubes in an acidic reaction condition and the formation of MOF‐5 square sheets under the basic reaction condition. Reproduced with permission.^[^ ^110^ ^]^ Copyright 2018, Elsevier.

MOFs exhibit a remarkable range of morphologies characterized by their distinct topologies, which regulate the structural arrangement and dimensionality of these materials.^[^ ^107^, ^108^ ^]^ As illustrated in **Figure** **6**a, the crystalline structure of MOFs affects their dimensionalities.^[^ ^109^ ^]^ The intrinsic crystallographic complexity of MOFs allows for tailored synthesis processes. Although it is feasible to design methods for synthesizing monodisperse particles, the formation of morphologies like rods and sheets often results in agglomeration. In such cases, the use of surfactants and specialized processing conditions becomes essential to prevent this agglomeration. Accordingly, the delicate coordination bonding within 3D morphologies can be adeptly transformed into 1D or 2D structures with precise primary processing.^[^ ^107^ ^]^

Achieving the desired morphology of MOFs mainly depends upon the addition of the particular additive or modulator during controlled synthesis.^[^ ^80^ ^]^ Additives suppress interpenetration by coordinating with metal clusters across pore windows. This interaction effectively alters crystal growth and overall morphology. Suresh et al. showed that by bridging metal clusters in specific directions through additives, such as aromatic dicarboxylates. The growth rates of different crystal facets of Zn_4_O‐based MOFs can be selectively controlled, resulting in the synthesis of morphologies with tailored crystallographic facets.^[^ ^80^ ^]^

**Figure 7:**
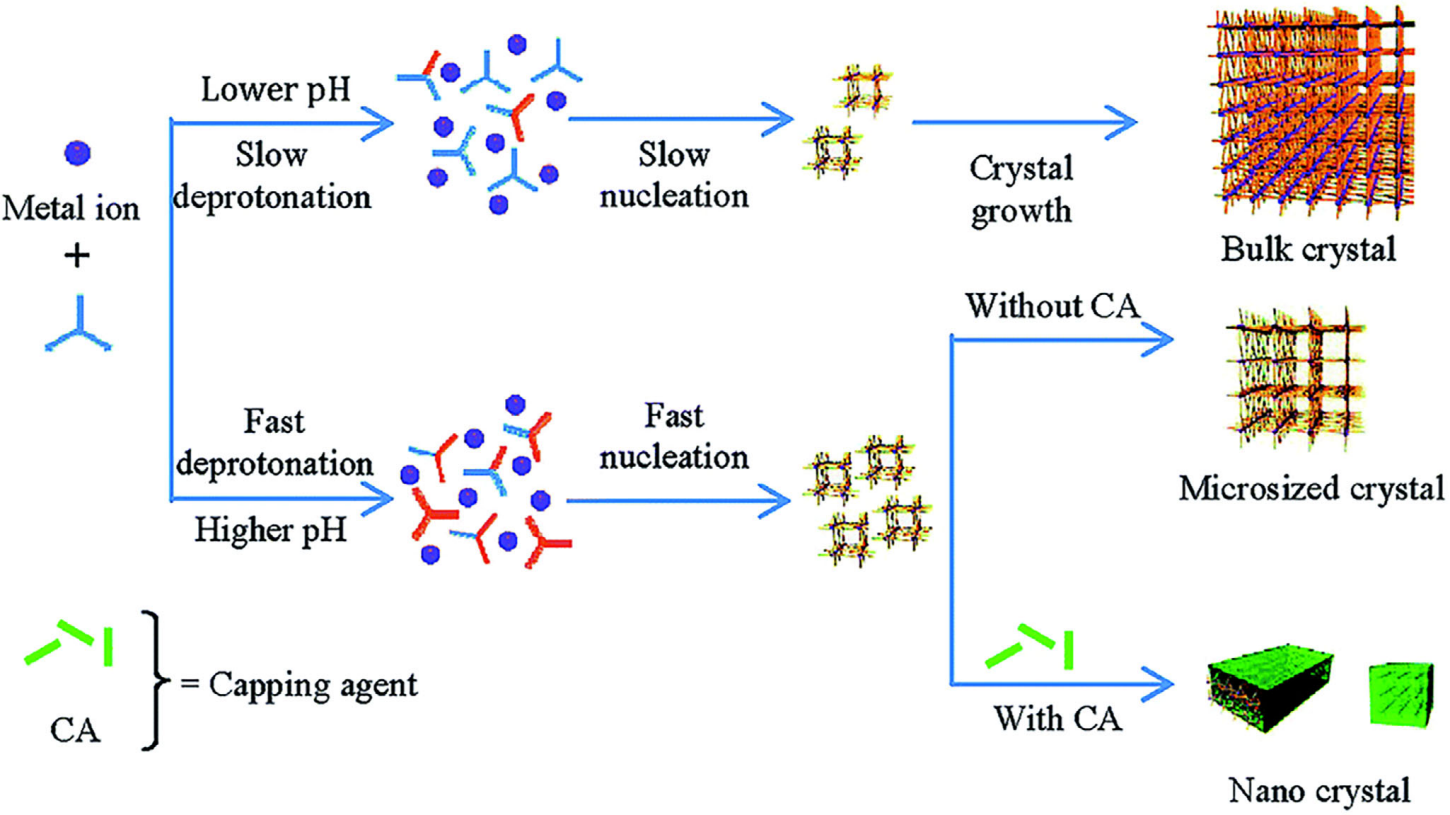
Emphasizing the dual effects of pH control and crystal capping during coordination modulation. Reproduced with permission.^[^ ^113^ ^]^ Copyright 2011, American Chemical Society.

Additives or capping agents can also influence the morphology of MOFs by altering the pH of the solvents.^[^ ^111^ ^]^ A pH can also be modulated using acidic and alkaline additives like acetic acid and ammonia, respectively, by adjusting the coordination environment of metal ions. Yuan et al. demonstrated that by tuning of pH using polyvinylpyrrolidone (PVP) and acetic acid, morphology can be transformed (Figure 6b).^[^ ^110^ ^]^ Delekar et al. synthesized square‐facet nanobars of Co‐MOF by tuning the pH of the precursor solutions using ammonia.^[^ ^112^ ^]^ The protonation and deprotonating effects from additives introduce protons into the system, affecting the coordination chemistry of ligands or metal clusters and crystal growth kinetics, ultimately shaping the final MOFs morphology (**Figure** **7**).^[^ ^113^ ^]^ Additionally, additives capable of hydrogen bonding interact with MOF components, directing the supramolecular assembly and influencing crystal growth processes.^[^ ^114^, ^115^ ^]^ However, additives can act as cosolvents or modify solvent properties.^[^ ^116^, ^117^ ^]^ The additive helps in controlling the solubility of the precursors and influences the nucleation as well as crystal growth, ultimately affecting the final morphology.^[^ ^118^ ^]^

**Figure 8:**
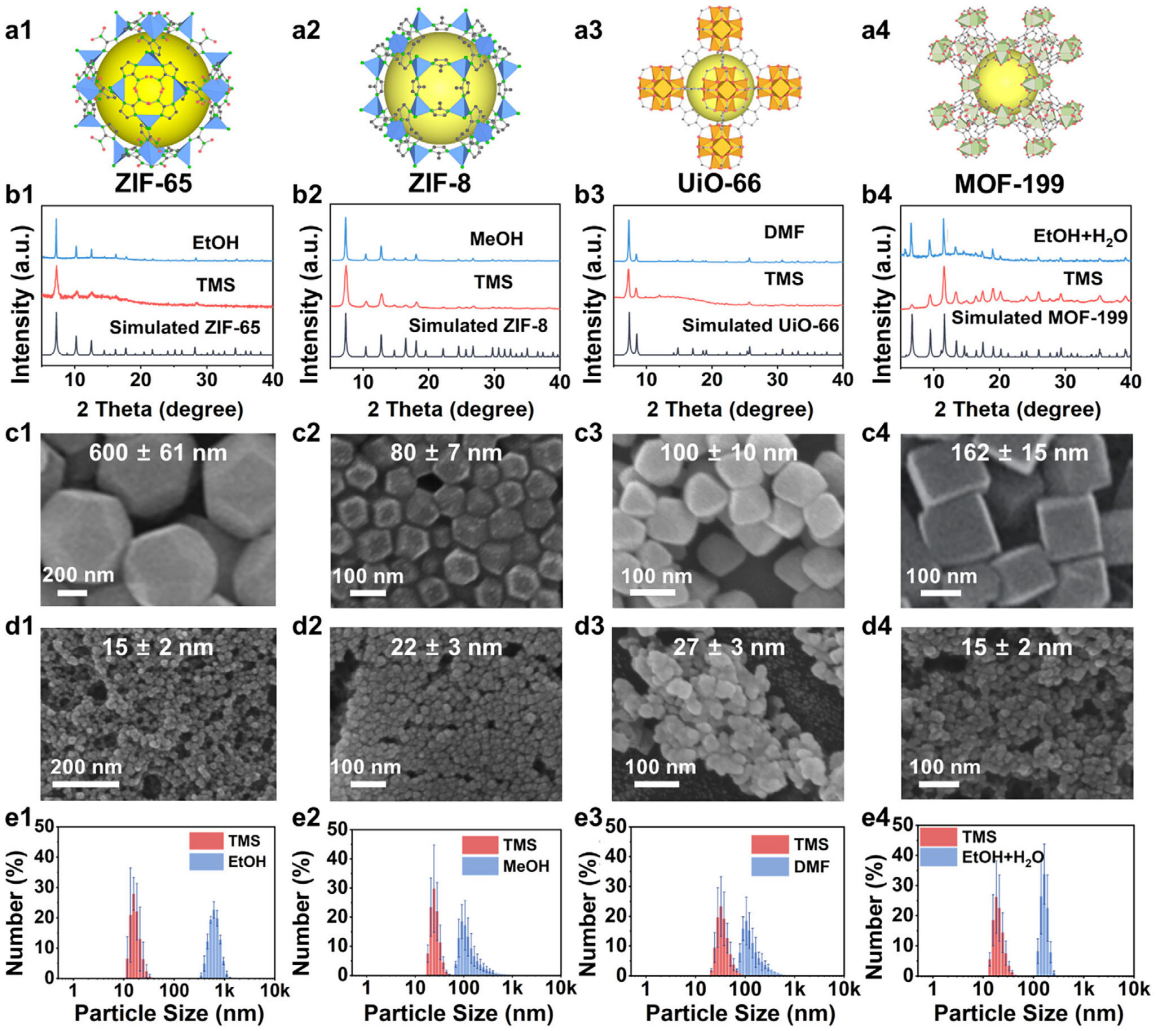
a1–a4) Topologies of synthesized MOFs. b1–b4) PXRD patterns of MOFs synthesized using various solvents. c1 to c4) SEM micrographs of MOFs synthesized using conventional solvents, and d1–d4) by using TMS. e1–e4) Particle size distributions of synthesized MOFs in various solvents as received by DLS analysis. Reproduced with permission.^[^ ^122^ ^]^ Copyright 2025, American Chemical Society.

Additionally, solvents play a pivotal role in synthesizing various MOFs.^[^ ^119^ ^]^ They affect the kinetics of reactions, reactant solubility, and stability of intermediates, thereby modifying the morphology, crystallinity, and overall qualities of MOFs.^[^ ^118^, ^120^ ^]^ Further, the choice of solvent also facilitates deprotonation of the ligand and metal ion coordination, which are important for controlling particle size during the synthesis of MOFs.^[^ ^121^ ^]^ In a recent study by Wang et. al explored the role of tetramethylene sulfone (TMS) solvent was explored for making a series of various MOFs, such as ZIF‐8, ZIF‐65, UiO‐66, and MOF‐199, achieving comparatively smaller particle sizes than those obtained with conventional solvents.^[^ ^122^ ^]^ The topologies of synthesized MOFs were displayed in **Figure** **8**a1–a4. The as‐synthesized ZIF‐8, ZIF‐65, UiO‐66, and MOF‐199 using TMS were confirmed by PXRD patterns, consistent with the standard report (Figure 8b1–b4). Remarkably, using TMS instead of other common solvents led to a severe reduction in particle size for ZIF‐8 (80 to 22 nm), ZIF‐65 (600–15 nm), UiO‐66 (100–27 nm), and MOF‐199 (162–15 nm), while maintaining higher yields (Figure 8c1–e4). Thus, the study underscores the role of the TMS solvent for producing nanoporous MOF nanocrystals with ultrafine dimensions.^[^ ^122^ ^]^

### 3.3 Role of MOFs as a Parent/Template Material

**Figure 9:**
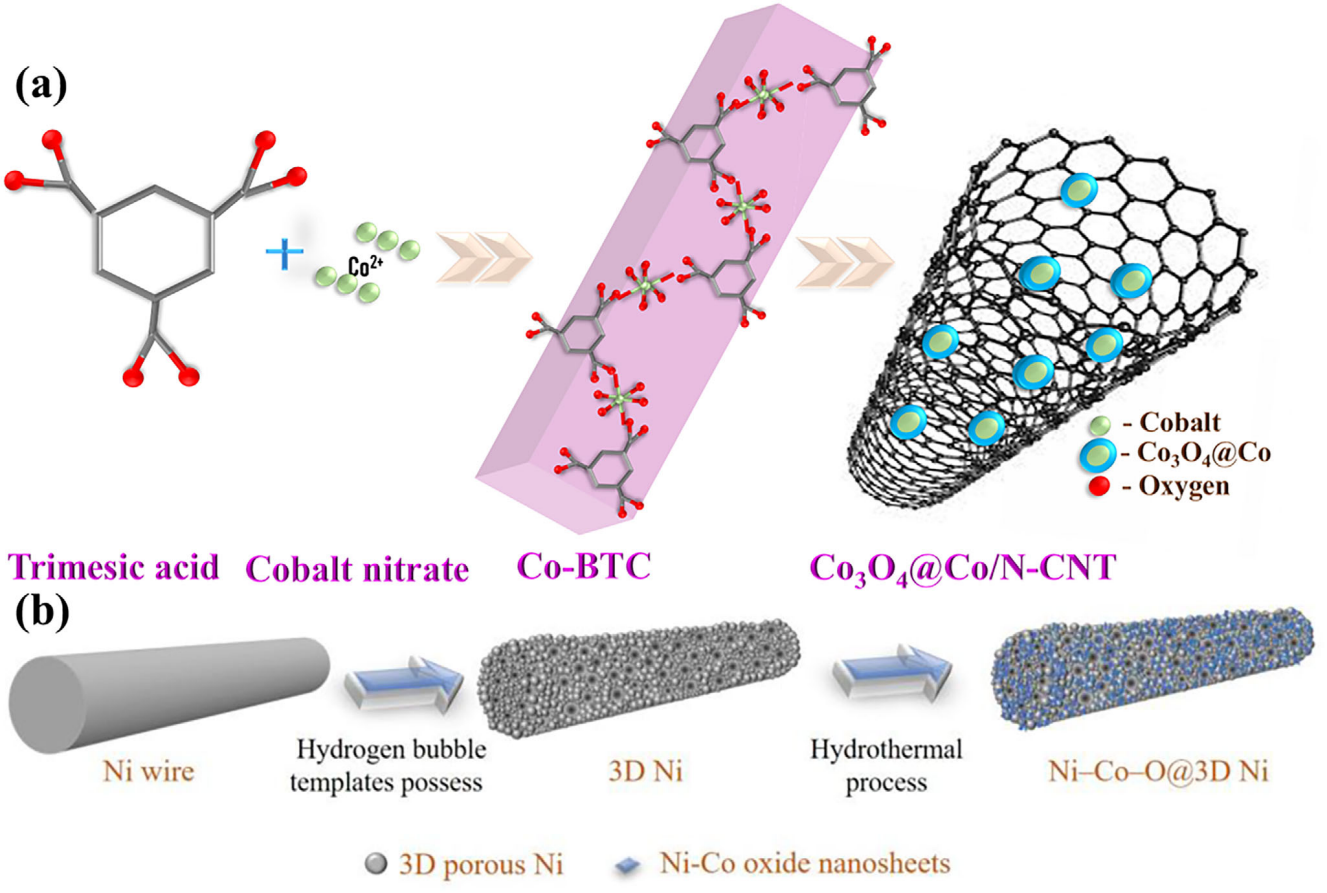
a) Schematic illustration of the synthesis of Co_3_O_4_@Co/N‐CNT. Reproduced with permission.^[^ ^112^ ^]^ Copyright 2022, American Chemical Society. b) Diagrammatic depictions of the synthesis of Ni‐Co‐O@3D Ni. Reproduced with permission.^[^ ^126^ ^]^ Copyright 2023, MDPI.

MOFs can serve as versatile templates for synthesizing nanostructures.^[^ ^118^ ^]^ Their porous structures and well‐defined pores facilitate the sacrificial growth of nanostructures, resulting in materials with controlled size, shape, and surface properties.^[^ ^118^ ^]^ The high surface areas and large pore volumes of MOFs are excellent hosts for guest molecules, capable of adsorbing gases, liquids, or ions. The host‐guest nature of materials is crucial for a range of applications.^[^ ^123^ ^]^ MOFs also enable the synthesis of hybrid materials by incorporating functional components such as polymers or inorganic NPs.^[^ ^124^ ^]^ When synthesizing microporous structures, different types of templates can be employed, including sacrificial, semi‐sacrificial, and non‐sacrificial templates.^[^ ^125^ ^]^ These templates can be polymers, silica, metals, metal oxides, zeolites, and layered materials. In one of the studies square‐facet Co‐MOF nanobar was used as a template for synthesizing Co_3_O_4_@Co/N‐CNT, a core–shell structure for SC applications (**Figure** **9**a).^[^ ^112^ ^]^

However, soft templates involve surfactant micelles, DNA‐like polymers, copolymers, ionic liquids, and colloidal suspensions as a scaffold for the vertical growth of MnS_2_‐CoS_2_ nanosheets. This enables the formation of a porous structure within the Mn‐Co‐S composite, enhancing its conductivity and stability.^[^ ^127^ ^]^ Through the template‐assisted synthesis, Mn‐Co‐S composite achieved a notable Cs of 1158.00 F g^−1^ at 1.0 A g^−1^. In most cases, the substrates used as templates have been proven to improve the functionality and uplift the performance of the resultant product. Chang et al. utilized a porous 3D Ni template, where Ni‐Co oxide nanosheets were integrated within a 3D porous Ni structure to create a binder‐free electrode for SCs.^[^ ^126^ ^]^ The synthesis involved hydrogen bubble templates with different applied voltages, resulting in variations in physicochemical properties that influenced the subsequent growth of Ni‐Co‐O nanosheets, as presented in Figure 9b.

## 4 MOF Derivatives and Their Supercapacitor Performance

### 4.1 MOF‐Templated Metal Oxides

A key drawback of pristine MOFs is a poor electrical conductivity caused by the insulating behaviour of the organic ligand.^[^ ^128^ ^]^ This limitation hinders their use in energy storage applications. To overcome these challenges, one can introduce other conducting materials in pristine MOFs, such as a carbonaceous matrix, metal oxide, etc.^[^ ^129^ ^]^ When metal oxides (MOs) lacking porous natures are used as an electrode active material for SCs, most of the mass acts as dead volume, and redox‐active sites remain unexplored.^[^ ^130^ ^]^ To fully exploit the MOs for excellent SC performance, MOs having well‐controlled hierarchically porous and textural characteristics should be achieved.^[^ ^131^ ^]^ The electrochemical SC performance of MOs as electrode materials depends upon surface area, crystallinity, porosity, surface energy, chemical reactivity, redox active sites, i.e., surface properties of the materials.^[^ ^132^, ^133^, ^134^ ^]^

There are various chemical routes for the synthesis of MOs, but challenges remain in achieving MOs with the desired physicochemical properties.^[^ ^13^ ^]^ Among these, MOF‐derived MOs (MOFs‐MOs) offer a facile way to enhance the performance of SC compared to pristine MOFs. MOFs‐MOs provide tunable composition, hierarchical pore structures, high surface area, higher redox active sites, and minimize the possibility of dead volume.^[^ ^135^ ^]^ Usually, MOFs‐MOs synthesised under controlled atmosphere retain the shape and porosity of the parent MOFs, having a high surface area.^[^ ^77^ ^]^ In connection with this, thousands of different functional MOF‐MOs have been reported to date. Some of the common MOF‐MOs are cobalt oxide (Co_3_O_4_),^[^ ^133^, ^136^, ^137^, ^138^, ^139^ ^]^ manganese oxide (Mn_2_O_3_),^[^ ^140^, ^141^ ^]^ nickel oxide (NiO),^[^ ^141^ ^]^ cerium oxide (CeO_2_),^[^ ^142^ ^]^ etc., obtained from their respective parent MOF with their achieved SC performance are summarised in **Table** **2**. Dai et al. demonstrated a straightforward interfacial‐engineering approach to synthesize oxygen‐vacancy‐rich Co_3_O_4_ nanosheets derived from Co‐MOFs.^[^ ^143^ ^]^ The optimized v‐Co_3_O_4_/carbon cloth material with oxygen vacancies that retain parent morphology and provide a deeper electrolyte penetration, enabling a high specific capacity of 414.0 C g^−1^ as mentioned in Table 2.

**Table 2:** Representative MOF‐Templated Metal Oxide Nanostructures with SC parameters.

Metal oxide	MOF precursor used	Electro‐lyte	Current density/ Scan rate [A g^−1^]	Specific Capacitance [F g^−1^]	Retention (%)/ Capacitance	Refs.
NiO	Ni‐MOF	KOH	1.0	28.5 mAhg^−1^	78.00/1000	[141]
NiO	Ni‐MOF	KOH	0.5	473.0	94.00/3000	[144]
NiO	Ni‐MOF	KOH	1.0	322.0	100.00/400	[145]
ZnO	ZIF‐8	KOH	1.0	292.0	–	[146]
Co_3_O_4_	ZIF‐67	–	1.0	140.0	97.03/5000	[147]
Co_3_O_4_	Co‐MOFs	LiOH	1.0	414.0 C g^−1^	73.9/15000	[143]
Co_3_O_4_	ZIF‐67	KOH	5.0	190.0	71.42/5000	[136]
Co_3_O_4_	Co‐MOF	KOH	1.0	1121.0	98.01/6000	[137]
Co_3_O_4_	ZIF‐67	KOH	1.0	1216.4	86.04/8000	[138]
Co_3_O_4_	Co‐MOFs	KOH	1.0	150.0	100.00/3400	[139]
Co_3_O_4_	ZIF‐67	KOH	5 mV s^−1^	504	–	[133]
MnO_X_	Mn‐MOF	Na_2_SO_4_	1.0	150.00	–	[140]
Mn_2_O_3_	Mn‐BTC MOF	Na_2_SO_4_	0.2	96.03	–	[148]
CeO_2_	Ce‐BTC MOF	KOH+ K_4_Fe(CN)_6_	1.0	779	91.00/10000	[142]

**Figure 10:**
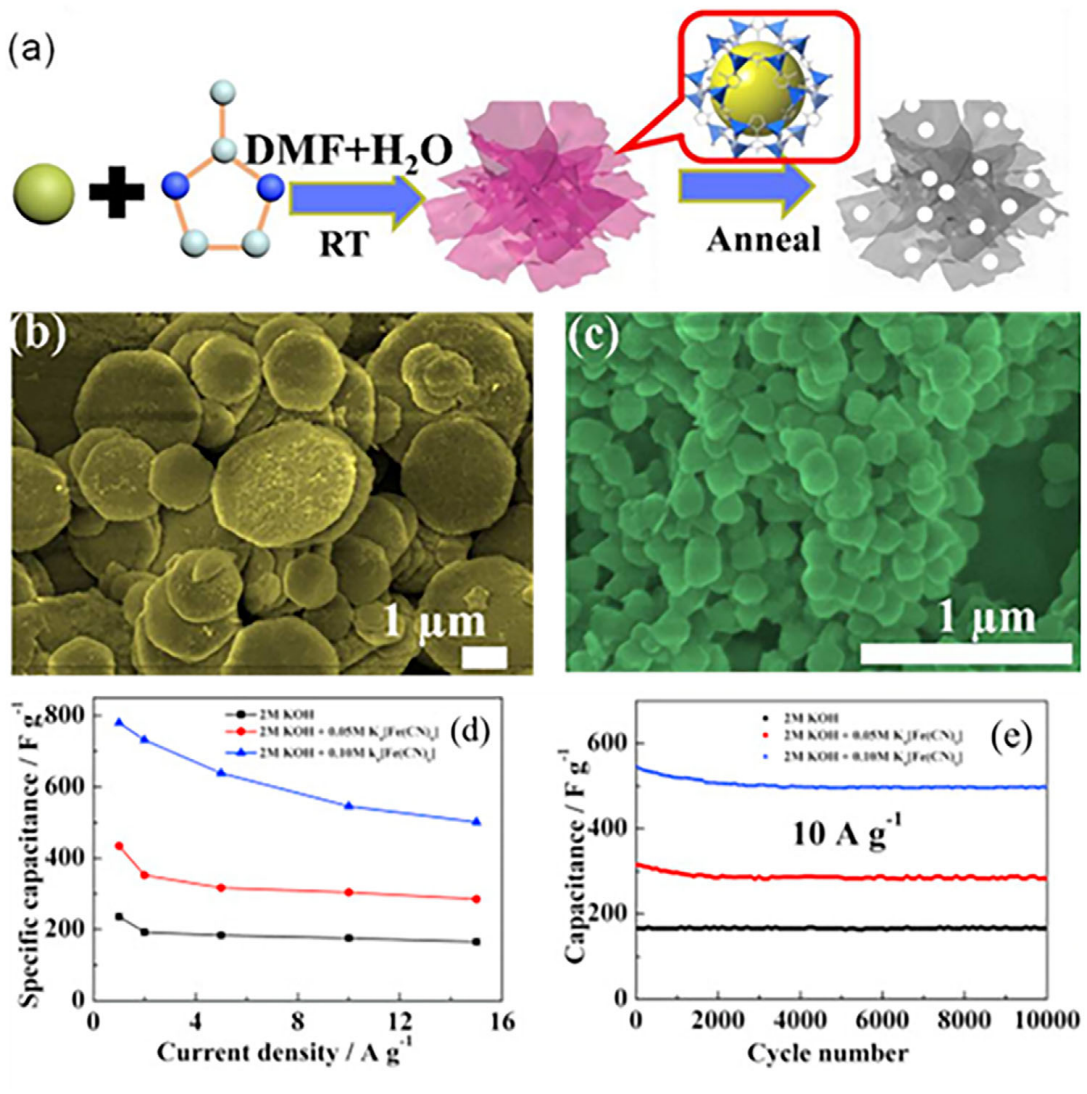
a) A schematic representation for the preparation of M‐Co_3_O_4_ ultrathin nanomeshes, b,c) SEM images of the W‐Co_3_O_4_ and the D‐Co_3_O_4_, respectively. Reproduced with permission.^[^ ^138^ ^]^ Copyright 2018, American Chemical Society. d) Variation of Cs with increasing Cd, e) Cycling stability behavior of CeO_2_. Reproduced with permission.^[^ ^142^ ^]^ Copyright 2016, Elsevier.

High‐performing electrode materials are the backbone of the SC applications. Particularly, TMOs such as Co_3_O_4_‐based functional materials are the better choice for SC applications due to their excellent electrochemical properties, including high theoretical capacitance, good electrical conductivity, and redox behavior.^[^ ^135^, ^149^ ^]^ The controlled annealing of MOFs enables precise tuning of Co_3_O_4_ morphology, resulting in nanostructures with increased active sites for redox reactions, uplifting the electrochemical performance. Notably, in a study by Wei et al. the synthesis of ZIF‐67 nanosheets was reported by coordinating the cobalt metal ion with 2‐methylimidazole in DMF and H_2_O solvent, which can be converted into ultrathin nanomeshes of Co_3_O_4_ (**Figure** **10**a).^[^ ^138^ ^]^ The resulting nanomeshes were observed to retain the original shape and exhibit uniform porosity, as like parent template surface area of 70.0 m^2^ g^−1^, demonstrating a high Cs of 1216.4 F g^−1^ at 1.0 A g^−1^. The FE‐SEM images reveal that W‐Co_3_O_4_ derived from ZIF‐67 in deionized water forms thick microplates with a wide size distribution, (Figure 10b), while using DMF as a solvent result in 100–200 nm irregular NPs, as depicted in Figure 10c. Thus, the choice of solvent significantly impacts the microstructure, a notable aspect of this work, as DMF promotes homogeneous nanosheet morphology due to its polar aprotic nature.^[^ ^138^ ^]^

In addition to Co_3_O_4_, there are several choices of MOFs‐TMOs that have also been extensively employed in SC applications, as some of them are discussed in Table 2. Accordingly, Wei et al. synthesized the hierarchical dumbbell‐shaped CeO_2_ architectures derived from Ce‐BTC MOF at room temperature.^[^ ^142^ ^]^ The incorporation of K_4_Fe(CN)_6_ as a redox‐active additive in the KOH electrolyte significantly improves the capacitive performance. The study shows that adding K_4_Fe(CN)_6_ to a 2 m KOH solution significantly increased the Cs of CeO_2_ electrodes. Without K_4_Fe(CN)_6_, capacitance was 235 F g^−1^ at a Cd of 1 A g^−1^. With 0.05 m K_4_Fe(CN)_6_, this value raised to 434 F g^−1^, and for 0.10 m K_4_Fe(CN)_6,_ it further increased to 779 F g^−1^, respectively (Figure 10d). Additionally, the electrodes demonstrate excellent long‐term stability, retaining 100%, 93%, and 91% of their capacitance after 10000 cycles in 2 m KOH, 2 m KOH + 0.05 m K_4_Fe(CN)_6_, and 2 m KOH + 0.1 m K_4_Fe(CN)_6_ electrolytes, respectively, at 10 A g^−1^ (Figure 10e). Thus, indicating that K_4_Fe(CN)_6_ enhances capacitance and also boosts the cycle stability, highlighting a unique approach to electrolyte optimization for superior capacitance and stability.

In another study, mesoporous Mn_2_O_3_ nanobars were successfully synthesized by exo‐templating of Mn‐BTC MOF, involving calcination of the Mn‐BTC MOF for 3 h at 650 °C.^[^ ^150^ ^]^ Further, it demonstrated potential as anodes in lithium‐ion batteries and pseudocapacitor electrodes. The cyclic voltammetry (CV) studies of Mn_2_O_3_ nanobars exhibited an increasing current response with higher scan rates within the potential window of 0.0–0.4 V. The maximum Cs obtained using a standard three‐electrode system was 250 F g^−1^ at a Cd of 0.2 A g^−1^. An asymmetric supercapacitor (ASC) fabricated using commercial activated carbon (AC) as the counter electrode and synthesized Mn_2_O_3_ nanobars as the positive electrode can significantly enhance Ed by extending the potential window up to 1.4 V. The device achieved a high Cs of around 150 F g^−1^ at 0.2 A g^−1^, and a high Ed of 147 Wh kg^−1^ with a Pd of 1004 W kg^−1^.^[^ ^150^ ^]^

Incorporation of certain molecules, like organic modulators, surfactants, and polymers, into MOFs precisely controls the crystal growth. This controlled growth is responsible for the change in morphology. As a result, the morphology tuning of MOFs or their derivatives led to a change in their porosity, surface area, and stability, which further influences their SC performance. For example, a study by Song et al. synthesized spindle‐like Fe_2_O_3_ nanostructures from a PVP‐assisted solvothermal route using Fe‐MOF.^[^ ^151^ ^]^ In this approach, PVP acts as a morphology tuning agent as well as a crystal growth regulator. PVP modulates nucleation kinetics, suppresses agglomerations of particles, and directs the formation of uniform, size‐controlled, spindle‐like nanostructures. Additionally, after calcination, PVP decomposes to a thin carbon layer that further modifies electron transfer, ion diffusion, and conductivity, boosting the Cs of 96.8 mAh g^−1^ at 1 A g^−1^. Moreover practical applicability of the material was tested by assembling the ASC device demonstrated an Ed of 20.12 Wh kg^−1^ at a power density of 140 W kg^−1^.^[^ ^151^ ^]^

### 4.2 Bimetallic MOF‐Templated Metal Oxide Composites

Bimetallic MOF‐templated MO composites have demonstrated superior performance as compared to bare MOF or single MO nanostructures in SC applications due to synergistic multiple redox reactions and enhanced electrical conductivity as a result of the combination of two metal ions.^[^ ^17^, ^152^ ^]^ Moreover, the addition of a second metal ion within the framework further enhances the stability of pristine MOFs.^[^ ^153^ ^]^ For example, MOF synthesized using Zn (II) ions, particularly [Zn_2_(OCO)_4_], exhibited enhanced stability when Zn(II) ions were replaced with other metals like Ni^2+^ or Cu^2+^.^[^ ^153^, ^154^ ^]^ Additionally, the intrinsic poor conductivity of pristine MOFs can be overcome by incorporating a second metal within MOFs. Such bimetallic MOFs with improved conductivity and redox properties are highly beneficial for enhancing electrochemical performance in SCs.^[^ ^155^ ^]^ Some of these MOF‐derived binary metal oxides are summarized in **Table** **3**. Among these materials, NiCo_2_O_4_ is recognized for its potential in energy storage through the formation of oxyhydroxide.^[^ ^156^, ^157^ ^]^ The superior electrical conductivity, high theoretical capacitance (2005 F g^−1^), and ease of synthesises of spinel NiCo_2_O_4_ make it one of the highly explored materials for SCs applications.^[^ ^1^, ^158^ ^]^

**Table 3:** Representative MOF‐Derived Binary Metal Oxide with SC parameters.

MOF precursor used	Derived binary metal oxides	Electrolyte	Current density/ Scan rate [A g^−1^]	Specific capacitance [F g^−1^]	Retention [%]/ capacitance	Refs.
Ni‐Co MOF	NiCo_2_O_4_	KOH	5.0	188.0 mAh g^−1^	85.25/3000	[158]
NiCo‐BTC MOF	NiCo_2_O_4_	KOH	20.0 mA cm^−2^	22.9	–	[130]
Ni‐ZIF‐67	Ni_x_Co_3‐x_ O_4_	KOH	1.0	1931.0	69.5/5000	[159]
MOF‐74‐NiCo	Ni_x_Co_3−x_O_4‐1_	KOH	1.0	797.0	80.0/10000	[160]
Ni‐ZIF‐67	Co_3_O_4_/NiCo_2_O_4_	KOH	1.0	770.0	70.0/10000	[156]
CoFe MOFs	CoFe_2_O_4_	LiCl	0.2 mA cm^−2^	2467.6 F cm^−3^	88.0/10000	[161]
NiFe_2_ MOF	NiFe_2_O_4_	KOH	0.25	833.0	73.0/700	[162]
Zn‐Co MOF (JUC‐155)	ZnCo_2_O_4_	KOH	5 mV s^−1^	451.0	97.9/1500	[163]
Mn‐Co‐ZIF	MnCo_2_O_4_	KOH	1.0	1763.0	95.0/4500	[164]
Zn/Ni‐MOF	Zn_0.65_Ni_0.35_O	KOH	1.0	471.1	81.3/ 1000	[165]

**Figure 11:**
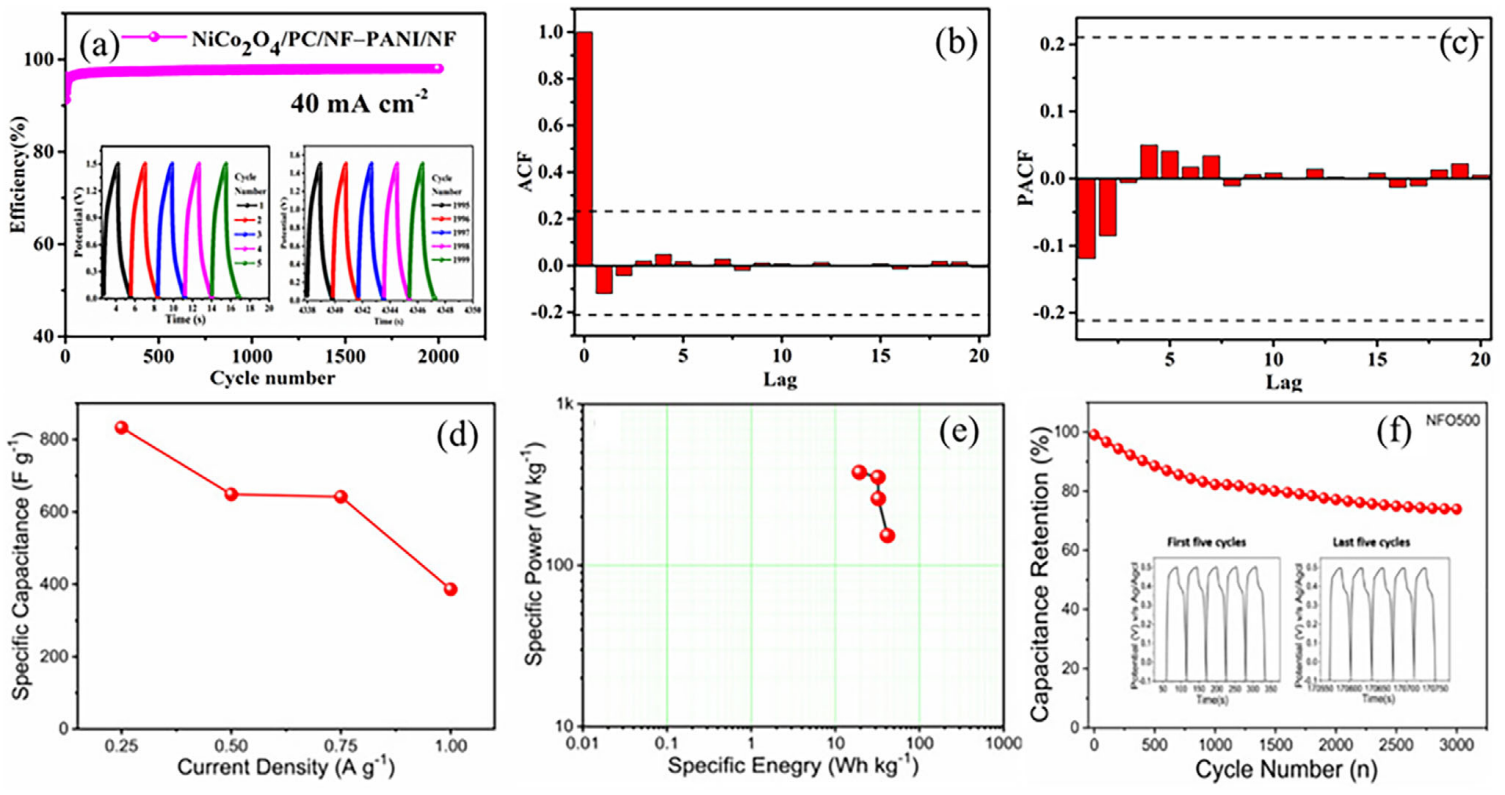
a) Cycle stability of NiCo_2_O_4_/PC/NF− PANI/NF with insets showing the first and last five cycles of 2000 continuous GCD cycles at 40 mA cm^−2^, b) ACF residuals, and c) PACF of residuals (the threshold bounds are shown by dashed lines). Reproduced with permission.^[^ ^130^ ^]^ Copyright 2024, American Chemical Society. d) The Cs of NFO500 against different Cds. e) Ragone plot of MOF‐derived NFO500. f) Plot of capacitance retention versus cycle number of NFO500 electrode. Reproduced with permission.^[^ ^162^ ^]^ Copyright 2021, Elsevier.

Delekar et al. used bimetallic NiCo‐MOF as a sacrificial template to derive NiCo_2_O_4_ with porous carbon (PC) for assembling both symmetric and asymmetric devices.^[^ ^130^ ^]^ The optimized anodes included NiCo_2_O_4_/PC, AC, and polyaniline (PANI) with aqueous electrolytes. By incorporating PANI as the anode material, the NiCo_2_O_4_/PC/NF−PANI/NF ASC device exhibited significantly higher Ed compared to other configurations, emphasizing the superior performance through optimization. **Figure** **11**a highlights the device's remarkable stability. The Holt–Winters Exponential Smoothing (HWES) technique reliably predicts the stability of the NiCo_2_O_4_/PC/NF−PANI/NF SC. The autocorrelation function (ACF) and partial autocorrelation function (PACF) values of the residuals, shown in Figure 11b,c, respectively, validate the aforementioned results. These findings underscore the importance of incorporating PANI as the anode and MOF‐derived porous NiCo_2_O_4_/PC as the cathode, demonstrating the practical applicability of the high‐performing SCs. The study integrated the device in series to power a red LED, showcasing the long‐term utilization potential, as analyzed through time series analysis (TSA) techniques.^[^ ^130^ ^]^

The synthesis of a series of Ni_x_Co_3‐x_O_4_ (isomorphic to Co_3_O_4_) crystal structure derived from MOF‐74 phases (MOF‐74‐Co, MOF‐74‐Ni, MOF‐74‐NiCo_1_, MOF‐74‐NiCo_2_, and MOF‐74‐NiCo_4_, with different Co: Ni ratios of 1:0, 0:1, 1:1, 2:1, and 4:1) was reported by Chen et al.^[^ ^160^ ^]^ The relationship between metal ratios in Ni_x_Co_3‐x_O_4_ and electrochemical performance is explored in their study. Ni_x_Co_3‐x_O_4‐1_ exhibited superior performance due to its higher surface area and abundant Ni metal ions. The Cs was observed to be 797 F g^−1^ at 1 A g^−1^ and retained 75% (597 F g^−1^) of the Cs value after 10000 cycles.^[^ ^160^ ^]^ Another similar work has been highlighted in Table 3, which details various Ni‐Co‐based MOF precursors, such as NiCo‐BTC MOF, and MOF‐74‐NiCo, Ni‐ZIF‐67, that are converted into their corresponding oxides NiCo_2_O_4_, Ni_x_Co_3‐x_O_4_, and Ni_x_Co_3−x_O_4‐1_ Co_3_O_4_/NiCo_2_O_4_.^[^ ^130^, ^156^, ^159^, ^160^ ^]^

Besides NiCo_2_O_4_, several other binary MOs composites are suitable for the SC applications.^[^ ^166^ ^]^ Subsequently, Patil et al. obtained the NiFe_2_O_4_ (NFO) samples by pyrolyzing solvothermally synthesized NiFe‐MOF.^[^ ^162^ ^]^ The successful synthesis of NFO through pyrolyzing at specific temperatures resulted in different morphologies, including threads, mesh‐like structures, and grains, showcasing the versatility in morphological control and their SC performance. The Cs values calculated from the galvanostatic charge discharge (GCD) curves as a function of the Cd are presented in Figure 11d. The electrochemical study of MOF‐derived NFO500 reported Cs of 833 F g^−1^ at 0.25 A g^−1^. The specific energy and the specific power of the NFO500 (NFO obtained by pyrolyzing at 500 ⁰C) electrode are calculated and are depicted in the Ragone plot, Figure 11e. GCD measurements were repeated for 3000 consecutive cycles at a Cd of 3.0 A g^−1^ in 1 m KOH to investigate the cyclic stability of the MOF‐derived NFO500 electrode, Figure 11f. NFO500 electrode retained 74.0% of its initial capacity after 3000 charge–discharge cycles with 84.0% of Coulombic efficiency. The remarkable supercapacitive performance of NFO500 electrodes is attributed to non‐sluggish charge transfer ability and mesh‐like structure, which allows electrolyte ions to infiltrate the electrode thoroughly.^[^ ^162^ ^]^

In a study by Dong et al. MnCo_2_O_4_ with nanocage morphology was synthesized through the calcination of bimetallic MnCo‐ZIF.^[^ ^164^ ^]^ As‐synthesized MnCo_2_O_4_ hollow polyhedrons demonstrated superior SC performance as compared to Co_3_O_4_ fabricated under similar conditions. MnCo_2_O_4_ exhibits a Cs of 1763 F g^−1^ at 1.0 A g^−1^, with 95.0% capacitance retention after 4500 cycles. Whereas, Co_3_O_4_ can achieve only Cs value of 1209 F g^−1^ with just retention of 81.2% of the initial capacity. Therefore, spinel MOs offer more active sites for redox reactions, leading to better electron conductivity. The hollow nanocage structure of MnCo_2_O_4_ allows proper shuttling and de‐shuttling of electrolyte ions. Thus, proving MnCo_2_O_4_ hollow polyhedrons to be a promising candidate for SC electrodes.

### 4.3 MOF‐Templated Carbonaceous Materials

MOF‐ templated carbonaceous materials often have additional exposed active sites, low density, and better interaction with the reaction medium compared to conventional carbonaceous materials.^[^ ^167^, ^168^ ^]^ Additionally, hollow structures of these carbonaceous materials improve stability by facilitating space for active material loading, providing pathways for diffusion, and buffering the volume variation that occurs during the reactions.^[^ ^169^, ^170^, ^171^ ^]^ Generally, pyrolyzing MOFs in an inert atmosphere yields carbonaceous materials.^[^ ^172^ ^]^ Also, pyrolyzing MOFs at higher temperatures transforms metal nodes and organic linkers into MOs and carbonaceous materials, respectively.^[^ ^173^ ^]^ Thereby, the removal of the MOs after itching yields porous carbons beneficial for SC application.^[^ ^174^ ^]^ The template MOFs determine the structure, porosity, and composition of the derived material, directly impacting its electrochemical characteristics.^[^ ^25^, ^175^ ^]^ Xu et al. first reported the preparation of nanoporous carbons (NPCs) from Zn‐MOF with furfuryl alcohol (FA) as a carbon source.^[^ ^176^ ^]^ The obtained Zn‐MOF‐derived carbon displays a high surface area and notable SC performance by undergoing electrostatic interactions. After Xu's studies, other research groups followed the concept of carbonization of MOFs for synthesizing porous carbon materials with different morphologies, porosities, and graphitization. Yan et al. carbonized three different MOF templates (HKUST‐1, MOF‐5, and Al‐PCP) under the same experimental conditions, and they were coded as MC‐Cu, MC‐Zn, and MC‐Al, respectively.^[^ ^177^ ^]^

The three carbonized materials showed different surface textures, pore sizes, and electrochemical performance. The MC‐Al (1103 m^2^ g^−1^) showed the highest surface area as compared to other MC‐Zn (420 m^2^ g^−1^) and MC‐Cu (50 m^2^ g^−1^). The surface properties of MC‐Al were observed to be beneficial for having higher Cs values of 232.8 F g^−1^ at 100 mA g^−1^ compared to MC‐Zn and MC‐Cu electrodes. These highly porous materials with high surface area and high porosity can easily infiltrate a large amount of electrolyte ions, leading to high capacitance and fast charge/discharge rates.^[^ ^184^ ^]^ Lim et al. also reported that MOFs with linkers of higher carbon density yield carbonaceous materials with lower surface area.^[^ ^178^ ^]^ Zhou et al. derived porous carbon with desired structural features, including morphology, porous structure, and crystallinity, using a CO_2_ infrared laser system.^[^ ^179^ ^]^ In addition, the role of metal species in achieving desired properties was also analyzed by studying various MOFs. **Table** **4**. summarises some more examples of MOF‐templated carbonaceous materials and analysis of their electrochemical performance for SCs. For instance, Ni‐based MOF‐templated approaches yield porous, conductive carbon nanostructures that improve charge transport and structural stability. Such as, NiO/rGO composite preserves high conductivity through the rGO matrix, NiO/C@CNF makes a self‐standing carbon support, and NiO/C/rGO hybrids develop stronger NiO‐carbon interfaces.^[^ ^180^, ^181^, ^182^ ^]^ Similarly, MOF‐templated carbonaceous materials have been utilized in other systems like Mn_3_O_4_@C, ZnMn_2_O_4_/C, rGO/Mn_3_O_4_, Mn_3_O_4_@N‐doped C, CeO_2_@C, and MnO/C, demonstrating excellent cycling stability and raised Cs up to 1102 F g^−1^, as highlighted in Table 4.^[^ ^183^, ^184^, ^185^, ^186^, ^187^, ^188^, ^189^ ^]^

**Table 4:** Representative MOF‐templated carbonaceous materials SC performance.

MOF precursor used	Derived carbon material	Electrolyte	Scan rate/ Current density [A g^−1^]	Specific capacitance [F g^−1^]	Retention (%)/ Capacitance	Refs.
MIL‐125 (Ti)‐derived	TiO_2_/C	H_2_SO_4_	5.0 mV s^−1^	258.0	95.0/2000	[190]
Ni‐MOF	NiO/rGO	KOH	1.0	435.2	49.34/25000	[180]
Ni‐MOF	NiO/C@CNF	KOH	1.0	742.2	90.42/ –	[191]
Ni‐MOF	NiO/C	KOH	1.0	496.0 C g^−1^	120.0/3000	[181]
Ni‐MOF	NiO/r‐GO	–	3.0	649.2 C g^−1^	81.01/5000	[182]
Co‐based MOFs (Co‐BDC)	Co_3_O_4_@Carbon	KOH	1.0	261.0	–	[183]
Mn‐based MOF	Mn_3_O_4_@C	Na_2_SO_4_	0.2	129.1	85.0/10000	[184]
Zn‐Mn‐MOFs	ZnMn_2_O_4_/carbon	Na_2_SO_4_	1.0	589.0	98.01/2000	[185]
Mn‐BTC	rGO/Mn_3_O_4_	–	0.5	420.0	–	[186]
ZIF‐67 MOF	Mn_3_O_4_@N‐doped C	KOH	1.0	980.0	97.77/5000	[187]
Ce‐MOF	CeO_2_@C	KOH	2.0	1102.0	–	[192]
Mn‐MIL‐100	MnO/C	Na_2_SO_4_	0.5	421.0	94.0/5000	[189]

**Figure 12:**
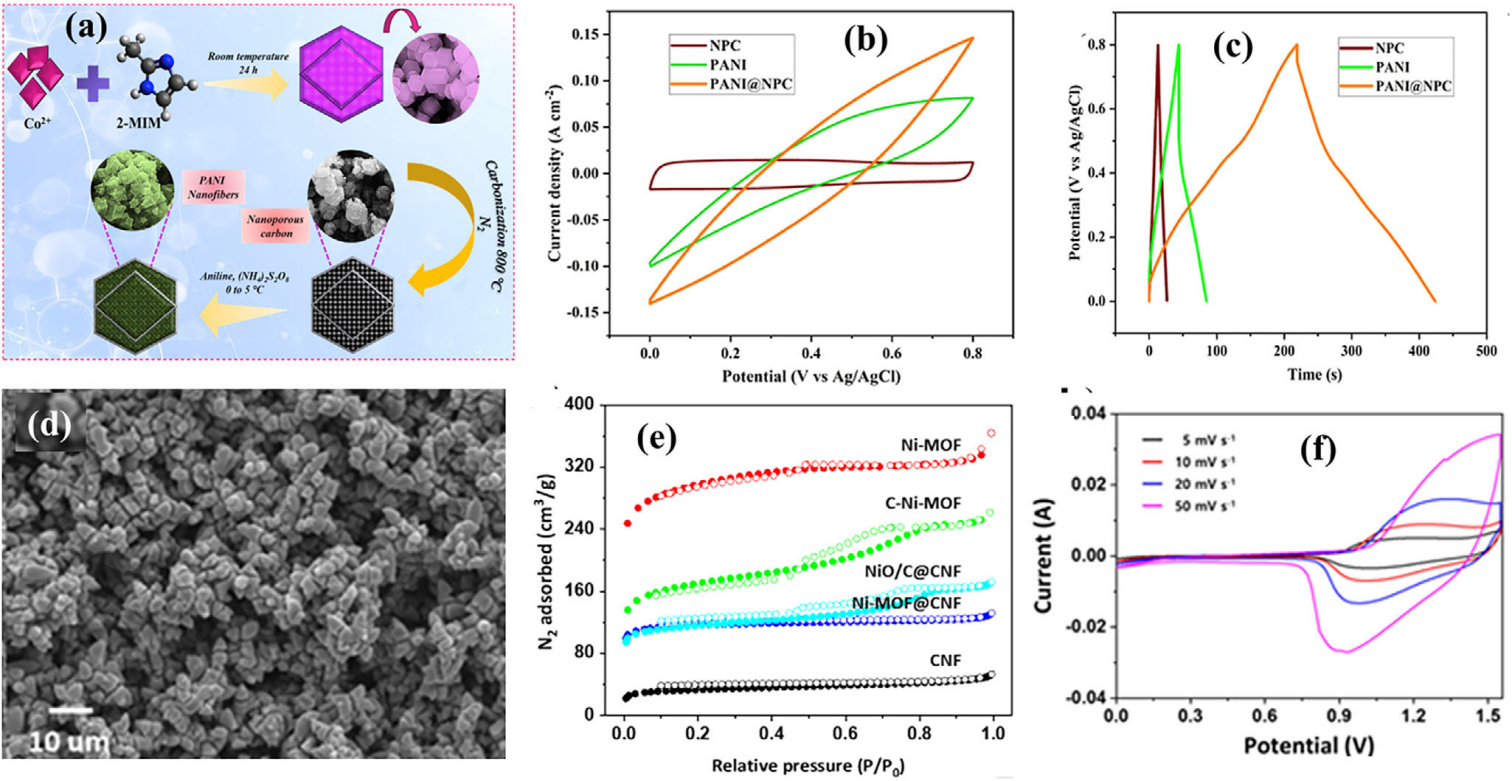
a) synthesis protocol PANI@NPC composite, b) comparative CV profiles at 100 mV s^−1^ of the NPC, PANI, and PANI@NPC composite, c) comparative GCD profiles at 10 mA cm^−2^ of the NPC, PANI, and ZIF‐67‐derived PANI@NPC composite. Reproduced with permission.^[^ ^193^ ^]^ Copyright 2025, Elsevier. d) FE‐SEM image of NiO/C@CNF, e) N_2_ adsorption‐desorption isotherms at 77 K. f) CV curves of the ASC at different potential ranges and at different scan rates. Reproduced with permission.^[^ ^191^ ^]^ Copyright 2021, Elsevier.

Shin et al. reported the synthesis and use of nickel oxide‐carbon composites obtained by directly carbonizing a Ni‐MOF@carbon nanofiber (Ni‐MOF@CNF) for the fabrication of a self‐standing electrode for SCs applications.^[^ ^191^ ^]^ The resulting NiO/C@CNF, obtained through the carbonization of Ni‐MOF@CNF, features evenly dispersed MOF‐derived NiO within the carbon matrix (**Figure** **12**d). This core–shell nature leads to an enhanced surface area and enhanced electrical conductivity (Figure 12e). The practical feasibility of NiO/C@CNF was assessed by fabricating an ASC device, which showed Cs of 742.02 F g^−1^ at 1.0 A g^−1^ and specific Ed of 58.43 Wh kg^−1^, with Pd of 1947 W kg^−1^. Nonetheless, the ASC device also maintained a consistent CV shape across all scan rates, indicating excellent rate capability (Figure 12f). However, the lower Ed is a main limitation of carbonaceous materials derived from MOFs. To address this issue, integrating conducting polymer with carbonaceous materials provides a better choice to further improve the electrochemical performance.^[^ ^193^ ^]^ Conducting polymers like polypyrrole (PPy), polyaniline (PANI), and PEDOT showcase high pseudocapacitance owing to their reversible redox properties and suitable electrical conductivity.^[^ ^194^ ^]^

However, their poor mechanical stability and limited cycle life limit standalone use.^[^ ^195^ ^]^ By combining conducting polymers with carbaneceous materials achieved from MOFs, a synergistic benefit of hybrid architecture is attained.^[^ ^196^, ^197^ ^]^ A carbon matrix provides a conductive backbone and mechanical support, hindering the polymer from structural degradation, while the polymer raises Cs through a Faradaic charge storage mechanism.^[^ ^198^, ^199^ ^]^ The resulting composites reveal high Ed and Pd, enhancing rate capability and improved cycling stability. For example, Kolekar et al. developed a ZIF‐67‐derived PANI@NPC composite, where nanoporous carbon (NPC) was achieved from the ZIF‐67 template, while PANI constitutes a core shell via oxidative polymerization (Figure 12a).^[^ ^193^ ^]^ This PANI@NPC composite combines the synergetic benefits of EDLC (through NPC) and pseudocapacitance (through PANI), providing a Cs of 1576 F g^−1^, an Ed of 42.3 Wh kg^−1^, and 82.22% capacitance retention after 5000 cycles. Figure 12b clearly showed the improved current response in CV and discharge time in GCD (Figure 12c) for PANI@NPCcomposite.

### 4.4 Functional Composites of MOF‐Derived Materials and Their Supercapacitor Applications

The SC performance mainly relies on various parameters such as high surface‐to‐volume ratio, crystallinity, chemical reactivity, etc.^[^ ^200^ ^]^ To date, various strategies have been employed to achieve controlled physicochemical properties. As MOF‐derived MOs can be attained with the desired composition, structures, and high surface‐to‐volume ratio. Still, it faces challenges like lower conductivity, structural stability issues, and surface areas that are higher but not up to the mark.^[^ ^201^, ^202^, ^203^ ^]^ So, combining MOF‐derived MOs with other materials such as carbonaceous materials, Noble metal NPs, as well as conductive polymers, to uplift physicochemical properties is under prime focus nowadays.^[^ ^204^, ^205^, ^206^ ^]^ Abdelhamid et al. developed Ni and Cu based MOs embedded into a carbon matrix (NiO/CuO@C).^[^ ^207^ ^]^ The NiO/CuO@C composite showed a high Cs of 1188 F g^−1^ over 1 A g^−1^ and good cycling stability, retaining over 93% of its capacitance after 5000 cycles. Such outstanding electrochemical performance was attributed to the uniform dispersion of metallic oxide within the conductive carbon matrices, providing numerous redox‐active sites and promoting rapid electron transport.^[^ ^207^ ^]^ In addition, doping or introducing heteroatoms like sulfur with MOF‐MOs provides a better choice to enhance the SC performance.^[^ ^208^, ^209^, ^210^ ^]^

**Figure 13:**
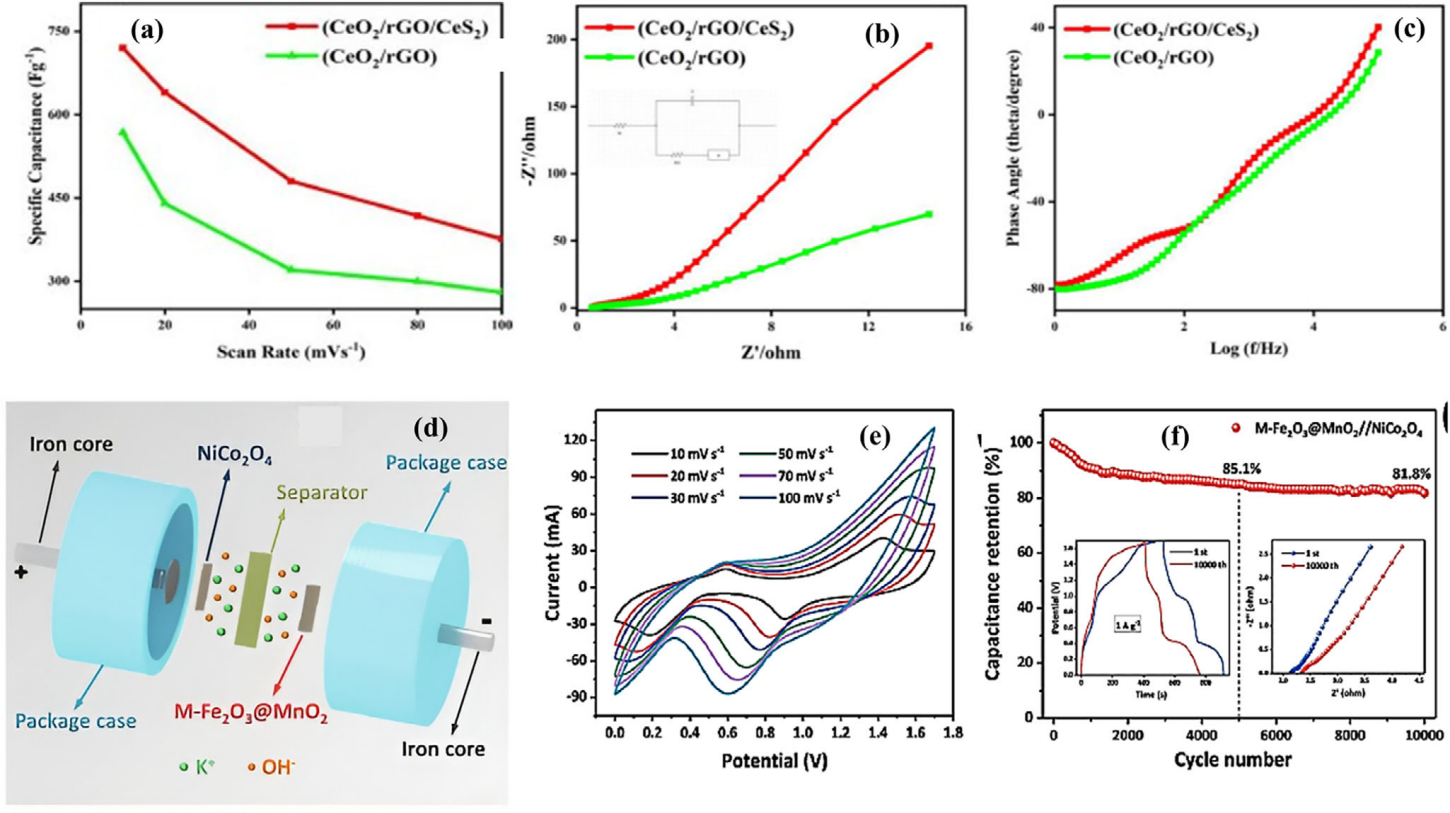
a) Scan Rate versus specific capacitance for CeO_2_/rGO and CeO_2_/rGO/CeS_2_. Electrochemical Impedance Spectroscopy (EIS) measurements, b) Nyquist plot CeO_2_/rGO and CeO_2_/rGO/CeS_2_. c) Bode plot for CeO_2_/rGO and CeO_2_/rGO/CeS_2_. Reproduced with permission.^[^ ^213^ ^]^ Copyright 2021, Elsevier. d) Schematic illustration of the assembled M‐Fe_2_O_3_@MnO_2_//NiCo_2_O_4_ HSC device. e) The CV curves of the HSC device at different scan rates, f) The Cycle stability of the HSC device at 5 A g^−1^. Reproduced with permission.^[^ ^152^ ^]^ Copyright 2021, Elsevier.

Doping enhances surface wettability between the electrode material and the electrolyte in carbon‐based compounds. And this further enhances the electrochemical performance by facilitating more active sites, particularly for pseudocapacitance.^[^ ^211^ ^]^ Accordingly, in one of the studies, hierarchical core–shell Co_3_S_4_@NiCo_2_O_4_ hollow nanosheet arrays were anchored on reduced graphene oxide/nickel foam (rGO/NF) via an MOF‐derived strategy.^[^ ^212^ ^]^ The 2D hollow Co_3_S_4_ nanosheets generated from MOFs can provide quick charge transport pathways and abundant electroactive sites. Coating the surface of Co_3_S_4_ with a material having a stable lattice and strong electrochemical activity can overcome the intrinsic flaws. The fabricated electrode Co_3_S_4_@NiCo_2_O_4_/rGO/NF showed improved cycling durability compared to Co_3_S_4_/rGO/NF due to enhanced mechanical stability by NiCo_2_O_4_. In another work, mesoporous Ce‐BTC MOF‐derived CeO_2_/rGO composite was initially synthesized and then subjected to sulfidation to yield CeO_2_/rGO/CeS_2_ nanocomposite.^[^ ^213^ ^]^ The testing of synthesized CeO_2_/rGO/CeS_2_ illustrates a Cs of 720.0 F g^−1^ at a scan rate of 10 mV s^−1,^ which was almost 1.25 times higher than for CeO_2_/rGO composite at the same scan rate, (**Figure** **13**a). Also, from Figure 13b,c, it was evident that CeO_2_/rGO/CeS_2_ nanocomposite exhibits lower series and charge transfer resistance. The high conductivity of CeS_2_ enhances electron transfer, while CeO_2_ contributes to charge transport. However, the electrostatic mechanism of rGO enhances the electrodes' electrochemical behavior. Govindan et al. developed a hybrid electrode material, CeO_2_/C/MoS_2_, derived from MOFs, integrated with molybdenum disulfide (MoS_2_) and carbon, for SCs.^[^ ^214^ ^]^ The CV curves of CeO_2_/C/MoS_2_//AC ASC, exhibiting hybrid EDLCs and Faradaic redox behaviors across different scan rates, which is attributed to the presence of carbon materials and the MoS_2_/CeO_2_ nanocomposite. Additionally, the superior stability of the CeO_2_/C/MoS_2_//AC ASC device can be attributed to the high structural stability of the MOF‐derivative, which promotes efficient ion transport and access to abundant electroactive sites, thereby extending charge–discharge cycles.

A study by the Yamauchi research group reported the synthesis of porous NiCo‐MOF‐74 as the starting precursor using a facile one‐step hydrothermal strategy.^[^ ^215^ ^]^ This NiCo‐MOF‐74 was heat treated at 800 and 350 ⁰C to yield graphitic carbon/Ni_x_Co_1‐x_ (in N_2_ atmosphere) and Ni_x_Co_1‐x_/Ni_x_Co_1‐x_O (in air atmosphere), respectively. Graphitic carbon/Ni_x_Co_1‐x_ (N_2_ atmosphere) showed a good Cs of 715.0 F g^−1^, which was higher than Ni_x_Co_1‐x_/Ni_x_Co_1‐x_O at a high Cd of 1.0 A g^−1^. These results are benefited by enhanced electrical conductivity enabled by graphitic carbon, low activation energies for multiple redox reactions, and the presence of mesopores for efficient electrolyte diffusion, leading to better exploration of electroactive sites.^[^ ^17^, ^215^ ^]^

Oh et al. utilized ZIF‐67 nanocrystals to fabricate highly porous Co_3_O_4_/NiCo_2_O_4_ nanostructures.^[^ ^156^ ^]^ The structure of the oxides was optimised by tailoring the crystallite size and pore structure of ZIF‐67 using the coordination modulation method. This Co_3_O_4_/NiCo_2_O_4_ nanostructure showed a maximum Cs of 770.0 F g^−1^ at 1.0 A g^−1^, with 70.0% retention of the initial capacitance after 10000 charge–discharge cycles.

Chen et al. successfully synthesized a pinecone‐like core–shell composite by growing MnO_2_ nanosheet arrays on M‐Fe_2_O_3_ [achieved by sacrificing the Fe‐MOF (MIL‐88A)].^[^ ^152^ ^]^ The M‐Fe_2_O_3_@MnO_2_ composite illustrated the Cs of 908.5 F g^−1^ and excellent cycle stability. The M‐Fe_2_O_3_@MnO_2_ composite was integrated into a HSC device with NiCo_2_O_4_, (Figure 13d), displaying distinct redox peaks indicative of its HSC with battery‐like characteristics seen from CV curves Figure 13e). Long‐term stability testing of this HSC revealed capacity retention of 85.1% and 81.8% after 5000 and 10000 cycles, respectively, as shown in Figure 13f. The capacity decay of the HSC device after continuous charge–discharge cycles is attributed to increased electrochemical and charge‐transfer resistance. Some representative examples of composites of MOF‐derived materials are summarized in **Table** **5**. It highlights various pristine, bimetallic, or hybrid MOFs templates like Ni/Co/Mn‐MOF, NiFe/Mn‐MOF, Fe‐MOF (MIL‐88A), and Mo‐POMs@Cu‐MOFs to achieve respective mixed MOs composites.^[^ ^152^, ^216^, ^217^, ^218^ ^]^ Among these composites, Co_3_O_4_/NiO/Mn_2_O_3_ resulting from CoNiMn‐MOF precursor showed the greatest Cs of 2809 F g^−1^ at 1.0 mA cm^−2^ along with 87.06% capacity retention after 10000 cycles, showcasing outstanding electrochemical stability and synergistically enhanced charge storage.^[^ ^219^ ^]^

**Table 5:** Composites of MOF‐Derived Materials and their supercapacitor performance.

Composites of MOF‐derived material	MOF precursor used	Electrolyte	Current density [A g^−1^]	Specific capacitance [F g^−1^]	Capacitance retention [%]	Refs.
NiMn_2_O_4_/CoMn_2_O_4_	Ni/Co/Mn‐MOF	–	3.0	756.4	92.01/3000	[216]
NiFeMn_2_O_4_	NiFe/Mn‐MOF	KOH	1.0	827.3	94.00/5000	[217]
M−Fe_2_O_3_@MnO_2_	Fe‐MOF (MIL‐88A)	KOH	1.0	908.5	78.07/8000	[152]
MoO_3_@CuO	Mo‐POMs@Cu‐MOFs	LiOH	1.0	86.3 mAh g^−1^	88.05/5000	[218]
Co_3_O_4_/NiO/Mn_2_O_3_	CoNiMn‐MOF	KOH	1.0 mA cm^−2^	2809.0	87.06/10000	[219]
M−Fe_2_O_3_@MnO_2_	Fe‐MOF (MIL‐88A)	KOH	1.0	908.5	78.07/8000	[152]
Fe_2_O_3_/NPC@Fe_3_C/EPCNFs	Fe‐MOFs	–	1.0	531.0	90.00/20000	[220]
CeO_2_/rGO/CeS_2_	Ce‐BTC MOF	KOH	2.5	720.0	–	[213]

## 5 Navigating Challenges and Future Directions

### 5.1 Current Limitations and Challenges

The well‐defined morphology and tunable chemical structures of MOFs are offering promising avenues for improving SC performance.^[^ ^221^ ^]^ MOFs present unique research opportunities that traditional carbon electrode materials cannot support due to their crystalline and tunable structures.^[^ ^222^ ^]^ These structures enable the exploration of how electrode design impacts SC performance and facilitates new mechanistic studies to understand charging mechanisms.^[^ ^223^ ^]^ Despite all these achievements, the performance of MOFs in SCs faces several challenges, including low potential windows, limited cycle lifetimes, lower conductivity in pristine MOFs, poor rate performances, high cost, and limited redox activity.^[^ ^221^, ^224^ ^]^

The size of the linkers in the MOFs is the key to deciding the charge transport mechanism.^[^ ^225^ ^]^ The lack of conjugation in the linker may hinder the transport of charge between it and metal centers. Further, MOF‐based electrodes are also observed to suffer from chemical instability in most of the aqueous electrolytes, losing active surface area.^[^ ^226^ ^]^ When the MOFs get degraded or collapse, their pore accessibility gets limited, increasing sluggishness. As already MOFs provide limited redox active sites as compared to the traditional TMOs. To address these challenges, researchers can employ suitable methods or modify traditional techniques to achieve the desired pore size, modulate framework dimensionality, include well‐defined functional groups, and leverage pseudo‐capacitive contributions.^[^ ^227^, ^228^ ^]^ Researchers are also focusing on creating MOF hybrids or derivatives to enhance MOFs electrochemical efficacy and structural stability.^[^ ^229^ ^]^

### 5.2 Strategies and Scope for Performance Improvement

MOFs hold potential beyond traditional applications. They are playing a key role in different heterogeneous applications such as catalysis, semiconductors, energy storage, and energy conversion devices.^[^ ^230^, ^231^ ^]^ The high specific surface area, tunable pore size, and controllable structure of MOFs make them excellent precursors or scaffolds for synthesizing metal‐based materials and composites.^[^ ^232^, ^233^ ^]^ There are several advantages of MOFs that can be further explored for SC applications by working on a few suggestions:

1. Wisely, choosing the ligands of MOFs where proper delocalization of electrons is possible with a few highly explored metal centers, like Co, Ni, Cu, and Fe.
2. In addition, further thermal treatments of the aforementioned MOFs lead to the formation of respective metal oxides having good theoretical capacitance.
3. Generally, MOF‐derived metal oxides show enhanced stability as compared to their parent MOF. This can be further improved by coating it with carbonaceous materials or polymers.
4. Optimization should also be carried out in case of electrode fabrication, such as electrode thickness as well as mass loading, for better infiltration of electrodes.
5. Scope also lies in the development of green and low‐cost synthesis routes for achieving MOFs and their derived materials with reduced costing and simplifying the complex synthesis techniques.
6. The synthesis of bimetallic MOF‐based materials can also provide multiple redox‐active sites.

By implementing these strategies, MOFs can be effectively integrated into energy storage devices, paving the way for high‐performance SCs and other advanced applications.

## 6 Impact and Applications

**Figure 14:**
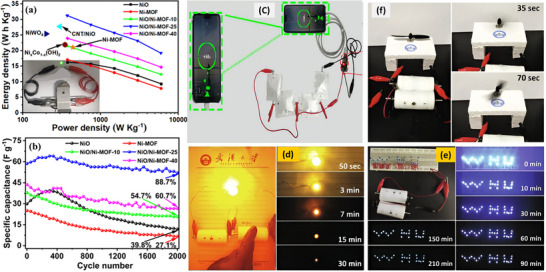
a) Ragone plot & a digital image of an LED light illuminated by two NiO/Ni‐MOF‐25//AC devices are included in the inset of a. b) cycle stability of the asymmetric SCs constructed with AC serving as the negative electrode and NiO/Ni‐MOFs, NiO, or Ni‐MOF as the positive electrode material at 8 A g^−1^. Reproduced with permission.^[^ ^237^ ^]^ Copyright 2021, American Chemical Society. c) mobile phone run by four CoSe_2_/NC‐400/AC units connected in series. Reproduced with permission.^[^ ^236^ ^]^ Copyright 2020, American Chemical Society. d) Digital images of a yellow LED lamp bead lit by two HSC devices in series. e) Digital images of 25 blue LED lights illuminated by two HSC devices in series. f) Digital images of an electric fan powered by two HSC devices in parallel. Reproduced with permission.^[^ ^152^ ^]^ Copyright 2021, Elsevier.

MOF‐based electrodes are promising candidates for advancing energy technologies such as lithium‐based batteries, SCs, hydrogen production, and energy conversion, as well as storage. Particularly, large specific surface areas, adjustable porosities, active sites, and rapid response make them ideal for practical energy storage solutions.^[^ ^234^, ^235^ ^]^ MOF‐derived SC electrode materials through practical applications reveal their potential for real‐time usage. Miao et al. connected four CoSe_2_/NC‐400//AC devices in series with a voltage regulator that successfully charged a mobile phone at a stable voltage of 5.0 V, showcasing their viability for potential application (see **Figure** **14**c). Delekar et al. fabricated a device, NiCo_2_O_4_/PC/NF‐PANI/NF SCs, capable of powering an LED for almost 17 s when two devices were connected in series. This practical test aligns well with the GCD results.^[^ ^130^ ^]^ Wang et al. fabricated the NiO/Ni‐MOF‐25//AC device showing the maximum Ed of 31.3 Wh kg^−1^ at a Pd of 374.2 W kg^−1^ (Figure 14a) and retained 88.7% of its initial capacitance after 2000 cycles (Figure 14b).^[^ ^237^ ^]^ Notably, two NiO/Ni‐MOF‐25//AC devices connected in series were assembled three months before the testing, which were capable of powering a green LED, demonstrating excellent stability of the device, see inset of Figure 14a. Chen et al. assembled an SC device with pinecone‐like M‐Fe_2_O_3_@MnO_2_ as the anode and urchin‐like NiCo_2_O_4_ as the cathode, which delivered a high Ed of 86.8 Wh kg^−1^ at 804.1 W kg^−1^.^[^ ^152^ ^]^ The device was capable of illuminating a “WHU” sign composed of 25 blue LEDs for over an astonishing 210 min, as presented (Figure 14d,e). Additionally, it powered an electric fan for 72 s, further highlighting its significant energy storage capacity as depicted in Figure 14f.

Ramasubramanian et al. synthesized nitrogen‐doped nanoporous carbon (NPC) and CNT/zirconium‐based MOF composites, which were employed as electrode materials in solid‐state SCs.^[^ ^238^ ^]^ The flexible SCs device achieved Ed and Pd of 0.0022 mWh cm^−2^ and 0.2 W cm^−2^, respectively, at a Cd of 0.4 mA cm^−2^, demonstrating its potential for powering strain sensors. The zirconia–carbon nanoporous material can be integrated into a strain sensor, enabling precise detection of mechanical deformations. These advancements in MOF‐derived materials and conductive hydrogels contribute to the integration of strain sensors powered by solid‐state SCs, paving the way for innovative wearable applications. These studies highlight the potential of MOF‐derived and related materials for practical SCs applications. Their ability to power various devices such as LEDs, mobile phones, power fans, strain sensors, etc, demonstrates significant advancements in energy storage technology.

Although MOFs and MOF derivatives are playing a crucial role in electrochemical energy storage devices, several issues must be addressed before large‐scale synthesis. Conventional MOFs suffer from drawbacks such as poor conductivity, low chemical stability, and structural integrity, limiting them for practical applications.^[^ ^17^, ^238^ ^]^ While the hybrids of MOFs and their derivatives can mitigate the aforementioned drawbacks, optimization is still needed for industrial applications. The scalability and cost endurance are key hurdles, as the currently explored synthesis strategies are solvent‐intensive and expensive. With developments in functionalization, scalable green synthesis, and stability enhancement, MOFs could become feasible materials for next‐generation sustainable energy systems.^[^ ^141^, ^239^, ^240^, ^241^ ^]^

## 7 Conclusion

MOFs and their derived materials show great potential for improving SC technology. MOFs possess highly tunable structures, and their base materials can follow EDLCs, a pseudocapacitive mechanism. In addition, MOF‐derived hybrid materials can follow both electrostatic and Faradic mechanisms, offering the potential for high capacitance and making them a promising alternative to traditional materials. However, several challenges, such as stability and conductivity, need to be addressed to fully harness this potential. MOF‐based SCs often exhibit lower stable potential windows, shorter cycle lifetimes, and slower charging rates compared to traditional porous carbon electrodes. These issues raise concerns about their practical use in commercial energy storage applications. Despite these challenges, the tunable characteristics of MOFs offer pathways to address these limitations. In this concern, refining the framework, dimensionality, surface chemistry, pore size, and proper choice of hybridizing materials are anticipated to lead to additional improvements. A deeper comprehension of charging and degradation processes is essential for advancing MOF‐based SCs. Although recent studies have started to reveal changes in electronic and ionic structures during charging, the mechanisms behind degradation remain largely unknown. The development of in situ experimental methods and molecular simulations is crucial for advancing MOFs applications. Furthermore, blending MOF hybrids and composites with other materials could improve both performance and stability. This knowledge will guide the design and synthesis of advanced materials, paving the way for the next generation of high‐performance SCs.

## Conflict of Interest

The authors declare no conflict of interest.

## Author Contributions

All authors have equally contributed to this review.

## Data Availability

No new primary research results have been included, and no new data were generated or analyzed as part of this review.
